# PP2A modulation overcomes multidrug resistance in chronic lymphocytic leukemia via mPTP-dependent apoptosis

**DOI:** 10.1172/JCI155938

**Published:** 2023-07-03

**Authors:** Kallesh D. Jayappa, Brian Tran, Vicki L. Gordon, Christopher Morris, Shekhar Saha, Caroline C. Farrington, Caitlin M. O’Connor, Kaitlin P. Zawacki, Krista M. Isaac, Mark Kester, Timothy P. Bender, Michael E. Williams, Craig A. Portell, Michael J. Weber, Goutham Narla

**Affiliations:** 1 Department of Microbiology, Immunology, and Cancer Biology, University of Virginia (UVA) School of Medicine, Charlottesville, Virginia, USA.; 2 Beirne B. Carter Center for Immunology Research, Charlottesville, Virginia, USA.; 3 Department of Pharmacology, Charlottesville, Virginia, USA.; 4 Division of Genetic Medicine, Department of Internal Medicine, the University of Michigan, Ann Arbor, Michigan, USA.; 5 Department of Biochemistry and Molecular Genetics, Charlottesville, Virginia, USA.; 6 Division of Hematology/Oncology, UVA School of Medicine, Charlottesville, Virginia, USA.; 7 Department of Biomedical Engineering, UVA, Charlottesville, Virginia, USA.; 8 Cancer Center, UVA, Charlottesville, Virginia, USA.

**Keywords:** Cell Biology, Oncology, Apoptosis pathways, Cancer, Phosphoprotein phosphatases

## Abstract

Targeted therapies such as venetoclax (VEN) (Bcl-2 inhibitor) have revolutionized the treatment of chronic lymphocytic leukemia (CLL). We previously reported that persister CLL cells in treated patients overexpress multiple antiapoptotic proteins and display resistance to proapoptotic agents. Here, we demonstrated that multidrug-resistant CLL cells in vivo exhibited apoptosis restriction at a pre-mitochondrial level due to insufficient activation of the Bax and Bak (Bax/Bak) proteins. Co-immunoprecipitation analyses with selective BH domain antagonists revealed that the pleiotropic proapoptotic protein (Bim) was prevented from activating Bax/Bak by “switching” interactions to other upregulated antiapoptotic proteins (Mcl-1, Bcl-xL, Bcl-2). Hence, treatments that bypass Bax/Bak restriction are required to deplete these resistant cells in patients. Protein phosphatase 2A (PP2A) contributes to oncogenesis and treatment resistance. We observed that small-molecule activator of PP2A (SMAP) induced cytotoxicity in multiple cancer cell lines and CLL samples, including multidrug-resistant leukemia and lymphoma cells. The SMAP (DT-061) activated apoptosis in multidrug-resistant CLL cells through induction of mitochondrial permeability transition pores, independent of Bax/Bak. DT-061 inhibited the growth of wild-type and Bax/Bak double-knockout, multidrug-resistant CLL cells in a xenograft mouse model. Collectively, we discovered multidrug-resistant CLL cells in patients and validated a pharmacologically tractable pathway to deplete this reservoir.

## Graphical abstract

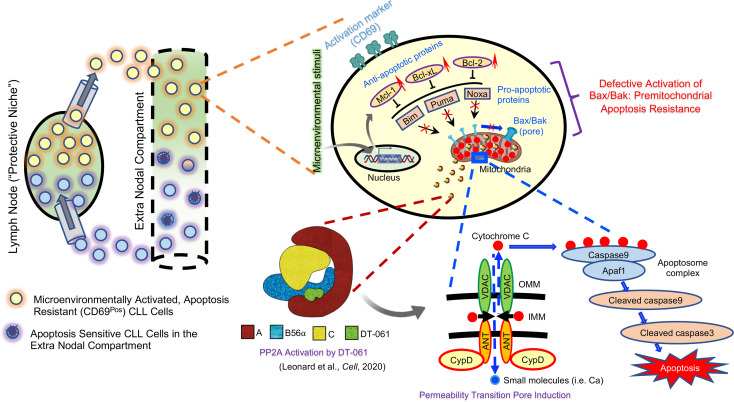

## Introduction

The introduction of proapoptotic pharmacological agents such as the antiapoptotic protein Bcl-2 inhibitor VEN has revolutionized the treatment of chronic lymphocytic leukemia (CLL) and mantle cell lymphoma (MCL). Although VEN as a single agent is broadly effective for CLL treatment (1, 2), the majority of the responses are incomplete. Moreover, most patients, including those experiencing complete clinical response, display drug-resistant, persistent cancer cells detectable by advanced molecular techniques (3). The de novo resistant cancer cells (i.e., found prior to treatment initiation) are a potential source for relapse (4). Consistently, patients displaying undetectable, persistent cancer cells (i.e., negative for minimal residual disease) frequently experience favorable long-term treatment outcomes (1, 5, 6).

The evidence suggests that microenvironmental interactions in vivo activate an antiapoptotic mechanism of resistance to VEN in CLL cells. This resistance is proposed to occur in the lymph node (LN) microenvironment (“protective niche”), where CLL cells encounter prosurvival signals, with recent data being consistent with this observation (1, 2, 4). Treatment with ibrutinib (IBR), an inhibitor of Bruton’s tyrosine kinase (BTK), is known to expel CLL cells from the protective LN in a subset of patients (7–9). We and others have tested IBR in combination with VEN in patients with CLL or MCL to exploit the therapeutic vulnerability generated by IBR-induced lymphocytosis in vivo (10–14), as well as the synergistic interaction of these agents ex vivo (15–17). Although clinical data with this combination show significant success in the majority of patients with CLL or MCL, a subset of patients display resistance even to this combination-based treatment (1, 2). Our recent study suggests that surviving persister cells in VEN-treated patients display resistance to a broad array of proapoptotic agents, including inhibitors of Bcl-2 (VEN), Mcl-1 (S63845), and Bcl-xL (A1155463) (4). At the molecular level, overexpression of multiple antiapoptotic proteins (Mcl-1, Bcl-xL, Bcl-2) establishes this multidrug resistance phenotype (4, 18). Thus, alternative treatments with novel mechanisms of action are required to deplete this antiapoptotic pool of multidrug-resistant cancer cells in patients.

Sustained activation of signaling pathways, either due to extrinsic cues, adoptive rewiring, kinase activation, or inactivation of phosphatases, contributes to cancer cell survival and drug resistance. While kinase inhibitors have been a major focus of targeted therapies, very little emphasis has been placed on phosphatase reactivation. Protein phosphatase 2A (PP2A), a serine/threonine phosphatase, is known to regulate key cellular functions such as cell growth, metabolism, and apoptosis (19). We recently identified a small-molecule activator of PP2A (*SMAP*) (20, 21), and we and others demonstrated that PP2A reactivation is a therapeutically viable strategy in various cancer models (22–29). Similar findings have been reported by modulating endogenous PP2A inhibitors such as CIP2A or SET (30). Given its broad regulatory function, PP2A reactivation holds enormous potential to overcome the multidrug resistance phenotype in cancer.

Here, we report that leukemic B cells with an activation phenotype in CLL patients displayed resistance to apoptosis at a pre-mitochondrial level and that this cell population was enriched during VEN treatment. Our protein interaction analyses revealed a potential resistance mechanism by which proapoptotic proteins (e.g., Bim) were prevented from activating Bax and Bak (Bax/Bak) by switching interactions with a series of upregulated antiapoptotic proteins, when cells were treated with selective BH domain antagonists. Our investigation showed that PP2A activation using a SMAP compound (DT-061) (21) induced marked cytotoxicity in leukemia cell lines or patient-derived CLL cells that exhibited antiapoptotic multidrug resistance. DT-061 triggered apoptosis in these cells via induction of permeability transition pores in the mitochondria (mPTPs), without engaging the classical Bax/Bak pathway. DT-061 was also well tolerated and effective at inhibiting the growth of multidrug-resistant CLL cells in vivo in a xenograft mouse model. Collectively, we report the existence of an antiapoptotic multidrug-resistant cancer cell population in patients with CLL and validate a pharmaceutically tractable pathway to deplete this reservoir.

## Results

### Leukemic B cells with an activation phenotype (CD69^Pos^) in patients with CLL display apoptosis resistance at a pre-mitochondrial level as a result of defective activation of Bax/Bak proteins.

Using ex vivo coculture systems, we and others have previously demonstrated that a variety of microenvironmental factors can induce overexpression of antiapoptotic proteins in CLL cells (15, 31–35). Phenotypically similar populations of CLL cells that overexpress Bcl-2, Mcl-1, or Bcl-xL have been recently detected in vivo (4, 18, 36–39). It is well established that these antiapoptotic proteins (Bcl-2, Mcl-1, Bcl-xL) functionally complement each other in restricting proapoptotic proteins that mediate Bax/Bak activation at a pre-mitochondrial level and the induction of apoptosis (40). Hence, microenvironmentally activated CLL cells (CD69^Pos^) that overexpress multiple antiapoptotic proteins could exhibit resistance to a series of proapoptotic agents as a result of insufficient activation of Bax/Bak proteins. Supporting this hypothesis, we observed that CLL cells activated by pretreatment with an agonist mix (CpG–ODN+sCD40L+IL-10) that upregulates the expression of multiple antiapoptotic proteins (15) displayed resistance to several proapoptotic agents including both inhibitors of Bcl-2, Mcl-1, and Bcl-xL and cytotoxic chemotherapeutic agents (Supplemental Figure 1, A–D; supplemental material available online with this article; https://doi.org/10.1172/JCI155938DS1). By performing flow cytometric analysis with anti-Bax (clone 6A7) and anti-Bak (clone NT) antibodies that recognize the active form of Bax and Bak proteins, respectively (41–43), we detected near-complete inhibition of Bax and Bak activation as well as subsequent post-mitochondrial steps (cleavage of caspase 9, caspase 3, and PARP) in CLL cells treated with proapoptotic drugs in the presence of an agonist mix ex vivo (Supplemental Figure 1, E–H; data not shown for chemotherapy agents). Thus, we suggest that microenvironmental activation can generate antiapoptotic multidrug resistance in CLL cells by restricting Bax/Bak activation at a pre-mitochondrial level.

**Figure 1:**
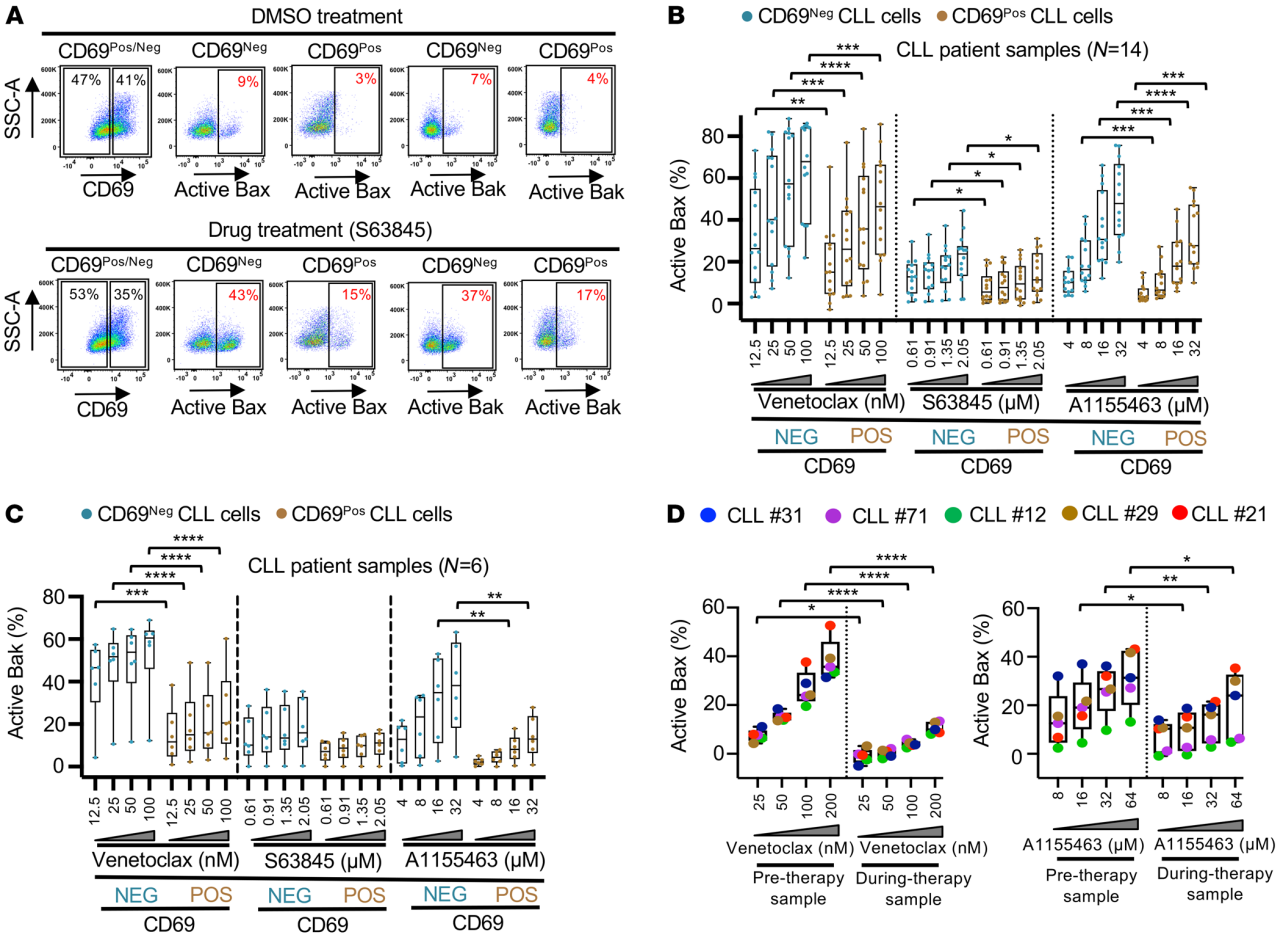
Circulating CLL cells with the CD69^Pos^ activation phenotype in vivo display pre-mitochondrial apoptosis restriction due to defective activation of Bax and Bak proteins. (**A**–**C**) Freshly frozen PBMCs from various CLL patients were screened in an ATA by incubation with an inhibitor of Bcl-2 (VEN: 12.5, 25, 50, 100 nM), Mcl-1 (S63845: 0.61, 0.91, 1.35, 2.05 μM), or Bcl-xL (A1155463: 4, 8, 16, 32 μM) for 3 hours without added agonists. (**A**) Representative flow images showing the expression of active Bax and Bak proteins in CD69^Pos^ and CD69^Neg^ CLL (viability dye^Neg^CD5^+^CD19^+^) cells in a patient’s PBMCs (patient [Pt] 08) incubated with DMSO or S63845 (2.05 μM). SSC-A, side scatter area. (**B** and **C**) Data showing the percentage of CD69^Pos^ or CD69^Neg^ CLL cells positive for active Bax (**B**) or active Bak (**C**) from multiple patient samples exposed to various proapoptotic agents in the ATA. (**D**) PBMCs from patients with CLL isolated prior to or during treatment with VEN (Supplemental Table 3) were analyzed in the ATA by incubation ex vivo with inhibitors of Bcl-2 (VEN: 25, 50, 100, 200 nM) or Bcl-xL (A1155463: 8, 16, 32, 64 μM) for 3 hours without agonists. Data show the percentage of CLL (viability dye^Neg^CD5^+^CD19^+^) cells positive for the active form of Bax following ex vivo incubation with VEN or A1155463. Statistical significance was determined by ANOVA with Šidák’s post hoc test for multiple comparisons. *P < 0.05, **P < 0.01, ***P < 0.001, and ****P < 0.0001. Data are presented as the mean ± SD.

In the recent study, we noted that microenvironmentally activated (CD69^Pos^) CLL cells in vivo display antiapoptotic multidrug resistance and that these cells are selectively enriched in patients undergoing treatment with VEN (4). To determine pre-mitochondrial apoptosis restriction in these microenvironmentally activated CLL cells in vivo, we assessed the activation of Bax/Bak in CD69^Pos/Neg^ CLL cells using an apoptosis threshold assay (ATA) following incubation of patient PBMCs with selective BH domain antagonists ex vivo (inhibitor of Bcl-2 [VEN], Mcl-1 [S63845], and Bcl-xL [A1155463]), as described in Methods. Our data demonstrated a significant reduction in Bax and Bak activation in CD69^Pos^ CLL cells as compared with their CD69^Neg^ counterparts in multiple patient samples incubated with inhibitors of Bcl-2 (VEN: 12.5, 25, 50, 100 nM), Mcl-1 (S63845: 0.61, 0.91, 1.35, 2.05 μM), or Bcl-xL (A1155463: 4, 8, 16, 32 μM) (Figure 1, A–C, and Supplemental Table 2). There was also a comparable reduction in cleaved caspase-9 (marker of post-mitochondrial apoptosis) expression in CD69^Pos^ CLL cells as compared with their CD69^Neg^ counterparts (Supplemental Figure 2), consistent with our earlier report (4). By performing a similar analysis on apoptosis-resistant persister CLL cells in patients undergoing treatment with VEN (4) (Supplemental Table 3), we noted that these cells were similarly impaired in Bax activation upon incubation with VEN, S63845, or A1155463 in an ATA ex vivo (Figure 1D). Together, these data suggest that microenvironmentally activated CLL cells in vivo were blocked from apoptosis induction at a pre-mitochondrial level and that this cell population was enriched in patients undergoing treatment with VEN.

### The proapoptotic protein Bim swapping by antiapoptotic proteins establishes pre-mitochondrial apoptosis restriction in multidrug-resistant CLL cells.

**Figure 2:**
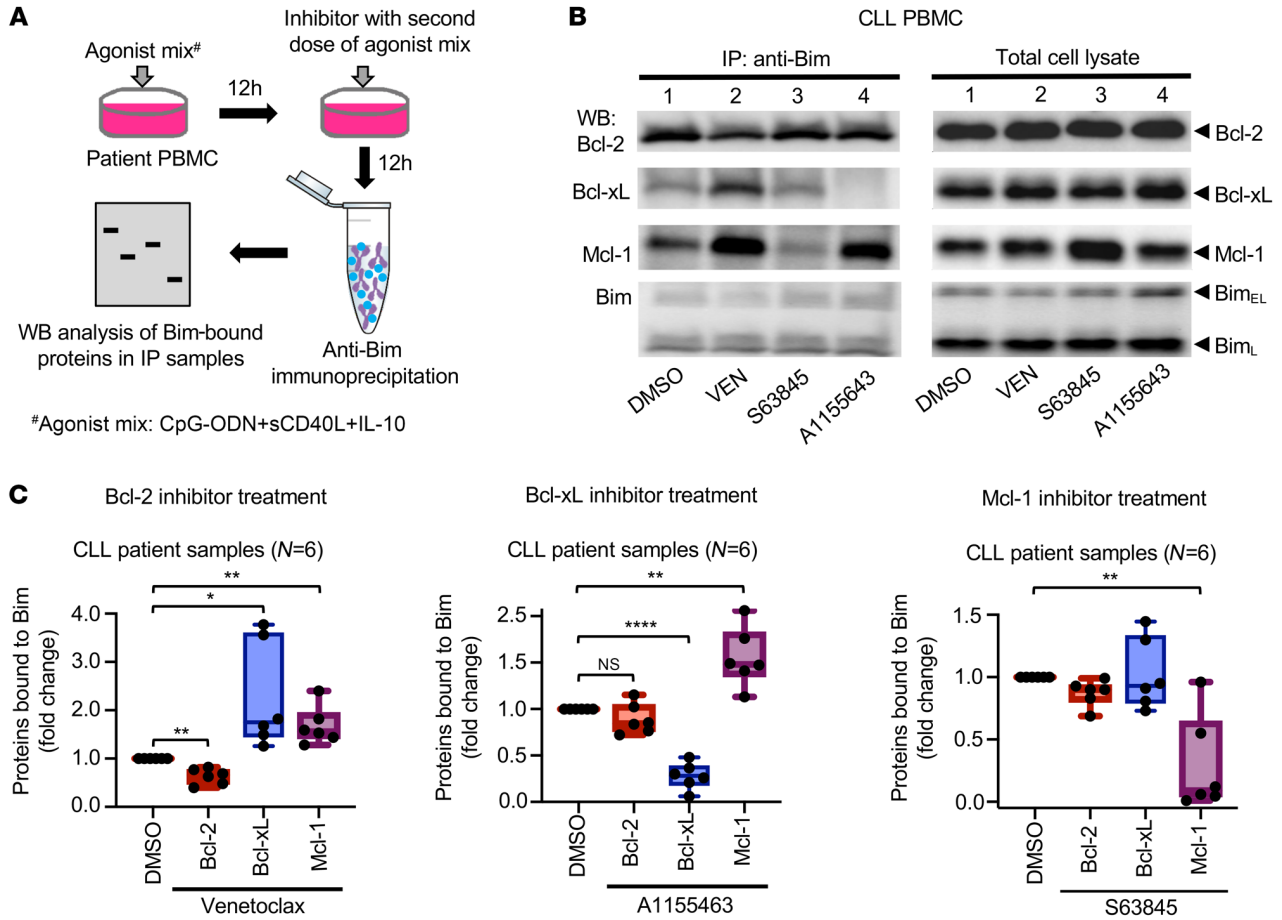
Proapoptotic protein Bim swapping by antiapoptotic proteins establishes pre-mitochondrial apoptosis restriction in multidrug-resistant CLL cells. (**A**) Diagram of the experimental design. PBMCs from patients with CLL were pretreated with the agonist mix for 12 hours to induce a multidrug-resistant state. Then, cells were treated with an inhibitor of Bcl-2 (VEN, 200 nM), Bcl-xL (A1155463, 16 μM), or Mcl-1 (S63845, 273 nM) as well as a second dose of agonist mix for an additional 12 hours. The proapoptotic protein Bim in cell lysates was immunoprecipitated using an anti-Bim antibody, and the antiapoptotic proteins Bcl-2, Bcl-xL, and Mcl-1 bound to Bim were analyzed by Western blotting using the corresponding antibodies. (**B**) WB images from a representative patient sample (Pt 33) showing a shift in Bim binding to Bcl-2, Mcl-1, and Bcl-xL in the presence of VEN, A1155463, or S63845 as compared with DMSO control. (**C**) Densitometric quantitation data from experiments involving 6 different patients with CLL demonstrating a shift in Bim recruitment to various antiapoptotic proteins in the presence of VEN, A1155463, or S63845 as compared with the DMSO control. Statistical significance was determined by Student’s *t* test. *P < 0.05, **P < 0.01, and ****P < 0.0001. Data are presented as the mean ± SD.

Since antiapoptotic proteins functionally complement each other in restricting proapoptotic protein function (40), we hypothesized that swapping of proapoptotic proteins by the upregulated antiapoptotic proteins “buffer” the generation of intrinsic apoptosis and enable generalized antiapoptotic multidrug resistance. To test this hypothesis, we assessed the recruitment of the proapoptotic protein Bim to Mcl-1, Bcl-xL, and Bcl-2 following incubation with BH domain antagonists (VEN, S63845, or A1155463) in agonist mix–treated primary CLL cells that exhibited overexpression of antiapoptotic proteins and multidrug resistance. This analysis was performed by examining co-immunoprecipitation of Bim with each of the antiapoptotic proteins, as described in Figure 2A. Our analysis with multiple independent CLL patient samples consistently demonstrated that higher levels of Bim were sequestered with Mcl-1 and Bcl-xL when cells were treated with the Bcl-2 inhibitor VEN, whereas the Bcl-2 interaction with Bim was reduced. Similarly, the Bcl-xL inhibitor A1155463 shifted Bim binding to Mcl-1, while the Bcl-xL interaction with Bim was reduced (Figure 2, B and C). Although treatment with the Mcl-1 inhibitor (S63845) significantly reduced Bim binding to Mcl-1, only a small fraction of Bim was sequestered by Bcl-xL, with substantial inter-patient variability noted. Nevertheless, these data collectively demonstrate that proapoptotic proteins were swapped between functionally redundant antiapoptotic proteins and that the sequestered proapoptotic proteins failed to activate the mitochondrial pore–forming proteins Bax and Bak, as demonstrated in Figure 1 and Supplemental Figure 1, E–H. We suggest that this mechanism underlies the antiapoptotic, multidrug-resistant phenotype observed in these cells. Together, these findings predicate that future therapies aimed at depleting this multidrug-resistant CLL cell reservoir must simultaneously block multiple antiapoptotic proteins or activate Bax/Bak-independent apoptosis or induce nonapoptotic cell death.

### The PP2A activation using small-molecule agonists induces cytotoxicity in leukemia/lymphoma cells that exhibit antiapoptotic multidrug resistance.

**Figure 3:**
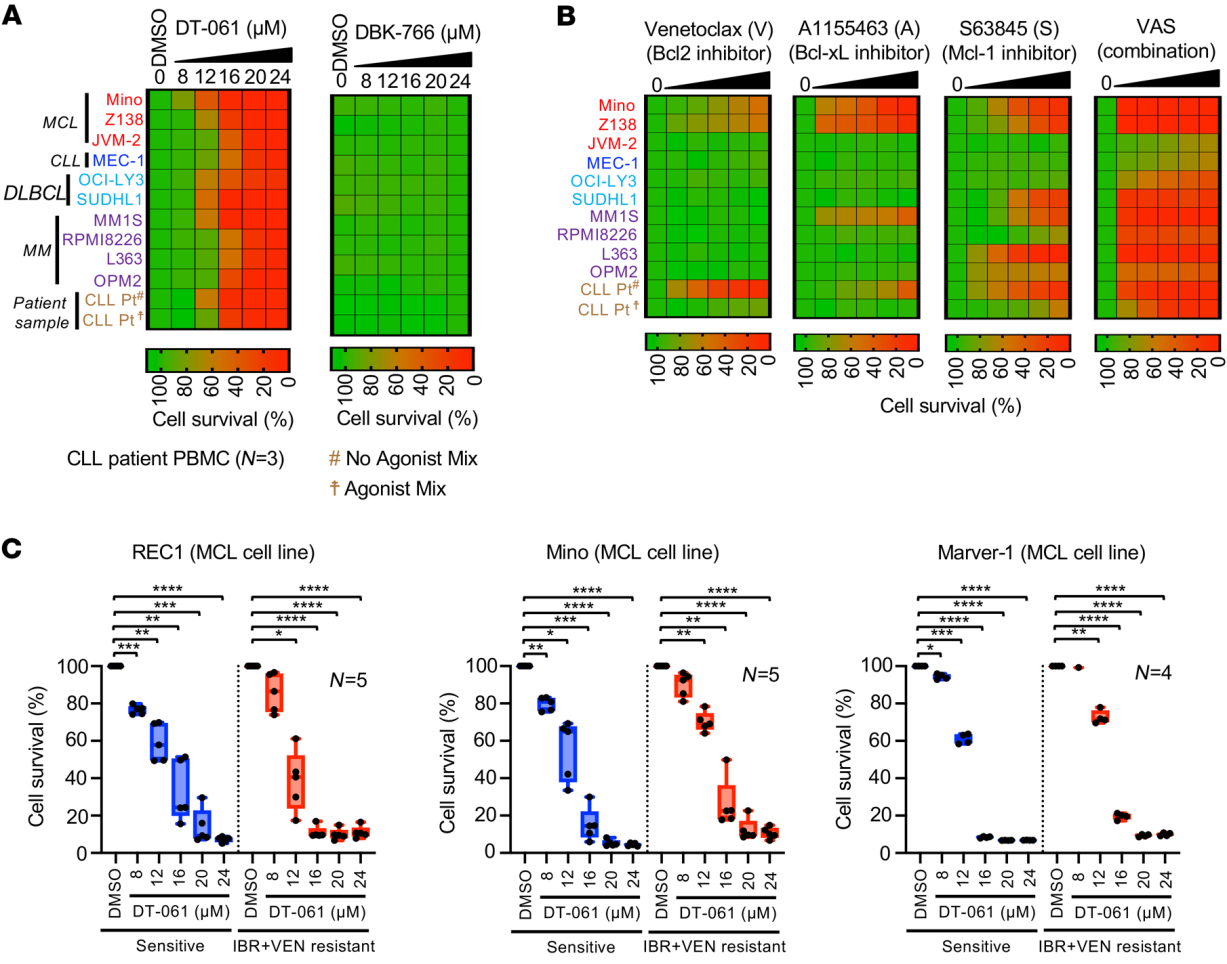
Activation of PP2A using SMAPs induces cytotoxicity in leukemia/lymphoma cells that exhibit apoptosis resistance. (**A**) Samples from patients with CLL were pretreated or not with the agonist mix, and various leukemia/lymphoma cell lines were analyzed for cytotoxicity with DT-061 or DBK-766 (8, 12, 16, 20, and 24 μM) using an alamarBlue assay. Samples were treated with drugs for 24 hours. The cytotoxicity data for cell lines were confirmed in 2 independent experiments, and multiple patient samples were screened. (**B**) Samples from patients with CLL were pretreated or not with the agonist mix as described in **B**, and various leukemia/lymphoma cell lines were analyzed for cytotoxicity with inhibitors of Bcl-2 (VEN; 12.5, 25, 50, 100, and 200 nM), Mcl-1 (S63845; 0.0625, 0.125, 0.25, 0.5, and 1 μM), Bcl-xL (A1155463; 0.5, 1, 2, 4, and 8 μM), and the combination (VAS) in an alamarBlue assay. Samples were treated with the drugs for 24 hours. The cytotoxicity data for the cell lines were confirmed in 2 independent experiments, and multiple patient samples were screened in an independent experiment. The average cell survival values are presented in a heatmap. (**C**) Sensitive or IBR- and VEN-resistant MCL cell lines were treated with DT-061 (8, 12, 16, 20, and 24 μM) for 24 hours, and drug-induced cytotoxicity was determined by alamarBlue assay. Statistical significance was determined by ANOVA with -Šidák’s post hoc test for multiple comparisons. *P < 0.05, **P < 0.01, ***P < 0.001, and ****P < 0.0001. The data were confirmed in multiple experiments as indicated and are presented as the mean ± SD.

We recently published on a series of first-in-class SMAPs (20, 21). We and others previously reported that PP2A activation is a viable therapeutic strategy against cancer (22–29). In one of the studies, we demonstrated that PP2A reactivation using a SMAP (TRC-382) is variably effective in a large number of cancer cell lines, including leukemia/lymphoma cell lines that exhibited relatively higher sensitivity (IC_50_ for solid vs. liquid cancers: 20.43 ± 7.45 vs. 14.98 ± 5.99 μM) (Supplemental Table 4) (29). Therefore, in this study we screened various B cell leukemia/lymphoma cell lines and CLL patient samples with a further pharmacologically optimized SMAP compound, DT-061, that has improved efficacy in vitro and in vivo. Additionally, we have since published the solved structure of the DT-061 molecule in complex with the PP2A-B56α holoenzyme (21). A pharmacologically inactive molecule, DBK-766 (TRC-766), that is still capable of binding but not activating PP2A was included as a negative control (22, 29). The results from the present study demonstrated that DT-061, but not DBK-766, was highly effective at inducing cell death in most tested B cell leukemia/lymphoma cell lines and CLL patient samples (Figure 3A). Strikingly, we found that DT-061 was equally effective in cell lines or patient-derived CLL cells that exhibited resistance to multiple BH domain antagonists (i.e., antiapoptotic, multidrug-resistant cells) (Figure 3B). We and others have previously reported the synergistic interaction between IBR and VEN in CLL and MCL (15–17). This combination treatment is now FDA approved or under clinical investigation in various types of leukemia and lymphoma, including CLL. Although the IBR and VEN combination is highly effective in the treatment of various leukemias and lymphomas, resistance to this combination treatment has been observed clinically. To assess SMAP activity in the resistance background, we generated IBR- and VEN-resistant MCL cell lines by exposing cells to the drug combination in culture for an extended duration (Supplemental Figure 3A). Our cytotoxicity data suggest that DT-061 was similarly effective in sensitive as well as resistant cell lines (Figure 3C) (IC_50_ [sensitive/resistant] for REC1, 15.4/11.7 μM; Mino, 13.0/13.6 μM; Marver-1, 12.4/12.9 μM). As expected, the resistant cell lines were least sensitive to the combination of IBR and VEN (Supplemental Figure 3, B–D).

Our previous study demonstrated that on-target modulation of PP2A using these small-molecule PP2A modulators drives the heterotrimerization of methylation-dependent PP2A subunits (21). Furthermore, we demonstrated using a validated PP2A methyl C–specific antibody that pharmacodynamic engagement of PP2A by these molecules can be measured in cellular and in vivo model systems (21). As expected, DT-061 induced the activation of PP2A in CLL cells, as measured by changes in methylation (Supplemental Figure 4A). As a further measure of PP2A activity, we observed dose-dependent AKT dephosphorylation, a well-documented PP2A substrate, in DT-061–treated CLL cells, which was effectively rescued by pretreatment with the serine/threonine phosphatase inhibitor calyculin A (Supplemental Figure 4, B and C).

Together, these results demonstrate that PP2A activation using a small-molecule agonist induced marked cytotoxicity in leukemia/lymphoma cells that exhibited antiapoptotic, multidrug resistance. Thus, PP2A activation can overcome antiapoptotic, multidrug resistance in leukemic B cells.

### The SMAP (DT-061) induces Bax/Bak-independent apoptosis in CLL cells.

**Figure 4:**
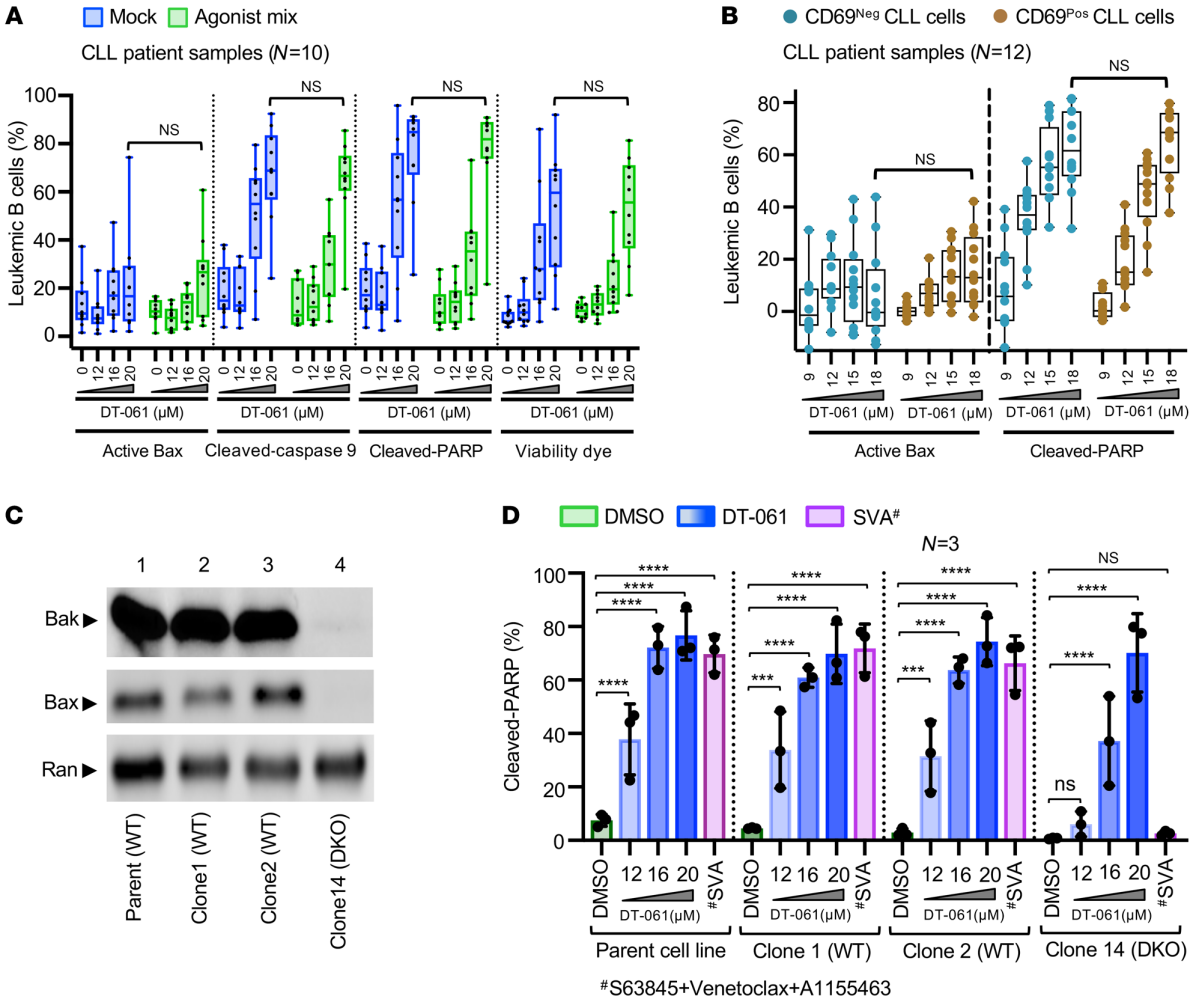
PP2A activation by the small-molecule agonist DT-061 induces Bax/Bak-independent apoptosis in CLL cells. (**A**) PBMCs from patients with CLL were preincubated with the agonist mix for 12 hours. Samples were treated with a second dose of the agonist mix as well as DT-061 (12, 16, and 20 μM) for 18 hours. Apoptosis induction (Bax activation and cleaved caspase 9 and cleaved PARP) and viability dye staining in CLL (CD5^+^CD19^+^) cells were analyzed by flow cytometry. (**B**) PBMCs from patients with CLL were screened by flow cytometry for Bax activation as well as cleaved PARP following incubation with DT-061 (9, 12, 15, and 18 μM) for 9 hours without added agonists. Data show the percentage CD69^Pos^ or CD69^Neg^ CLL (CD5^+^CD19^+^) cells positive for active Bax or cleaved PARP, after subtraction of the spontaneous apoptosis values from the DMSO-treated controls. (**C**) The Bax/Bak-DKO CLL cell line MEC1 was developed using the CRISPR/Cas9 system, as described in Methods. WB data show the expression of Bax and Bak proteins in WT and Bax/Bak-DKO clones. (**D**) The parent MEC1 cell line as well as WT and Bax/Bak-DKO clones were treated with DT-061 (12, 16, and 20 μM) or a combination of VEN (0.2 μM), S63845 (2 μM), and A1155463 (1.6 μM) (SVA) for 12 hours. Cleaved PARP was analyzed by flow cytometry. The average data from 3 independent experiments are presented as a bar graph, which shows the percentage of MEC1 cells positive for cleaved PARP. Statistical significance was determined by ANOVA with Šidák’s post hoc test for multiple comparisons. ***P < 0.001 and ****P < 0.0001. Data are presented as the mean ± SD.

Our above results demonstrate that the PP2A activator DT-061 was effective in killing multidrug-resistant leukemia/lymphoma cells. On the basis of this finding, we speculated that PP2A modulation overcomes antiapoptotic, multidrug resistance by either relieving Bax/Bak restriction or through the activation of an alternative cell death mechanism. To test this hypothesis, we performed flow cytometric analysis of Bax activation as well as of markers of post-mitochondrial apoptosis (cleaved caspase-9 and cleaved PARP) in agonist mix–treated primary CLL cells (as described in Supplemental Figure 1). To our surprise, DT-061 treatment drove a marked increase in cleaved caspase-9 and cleaved PARP expression in the absence of Bax activation in mock- as well as agonist mix–treated CLL cells (Figure 4A). Since we have previously demonstrated that microenvironmentally activated (CD69^Pos^) CLL cells in circulation exhibit de novo multidrug resistance (4), we examined apoptosis induction in CD69^Pos^ CLL cells upon DT-061 treatment in patient PBMC samples. Interestingly, DT-061 treatment induced apoptosis in the absence of Bax activation in CD69^Pos^ as well as CD69^Neg^ CLL cells (Figure 4B). These results suggest that PP2A modulation triggered apoptosis in multidrug-resistant CLL cells without engaging the classical Bax/Bak pathway, a known target of many proapoptotic anticancer therapies (44). To further validate this Bax/Bak-independent apoptotic phenotype, we generated a Bax/Bak double-knockout (DKO) CLL cell line, MEC1, using a CRISPR/Cas9 system. Because this genetic manipulation has not been successful in primary human CLL cells, we chose to generate these isogenic cell lines in the immortalized MEC1 CLL cell line. Consistent with our above observations, DT-061 induced marked cytotoxicity in Bax/Bak-DKO MEC1 cells, even though WT cells were slightly more sensitive to DT-061 treatment (IC_50_ for clone 1 [WT], 8.33 μM; clone 2 [WT], 8.27 μM; clone 14 [Bax/Bak-DKO], 10.67 μM) (Supplemental Figure 5A). Subsequently, we examined apoptosis induction in WT and Bax/Bak-DKO MEC1 cells treated with DT-061 by flow cytometric analysis of cleaved PARP. We found that DT-061 treatment induced significant apoptosis and a comparable loss of cell viability in both WT as well as Bax/Bak-DKO MEC1 cells (Figure 4, C and D, and Supplemental Figure 5B). As expected, the combination of BH domain antagonists (S63845+VEN+A1155463 [SVA]) that relies on the Bax/Bak pathway for apoptosis induction was ineffective in Bax/Bak-DKO, but not WT, MEC1 cells (Figure 4D and Supplemental Figure 5B). Together, these findings suggest that PP2A modulation using the SMAP (DT-061) induced Bax/Bak-independent mitochondrial apoptosis in CLL cells, a finding not previously reported to our knowledge.

### The SMAP (DT-061) triggers apoptosis in CLL cells by inducing mPTPs.

**Figure 5:**
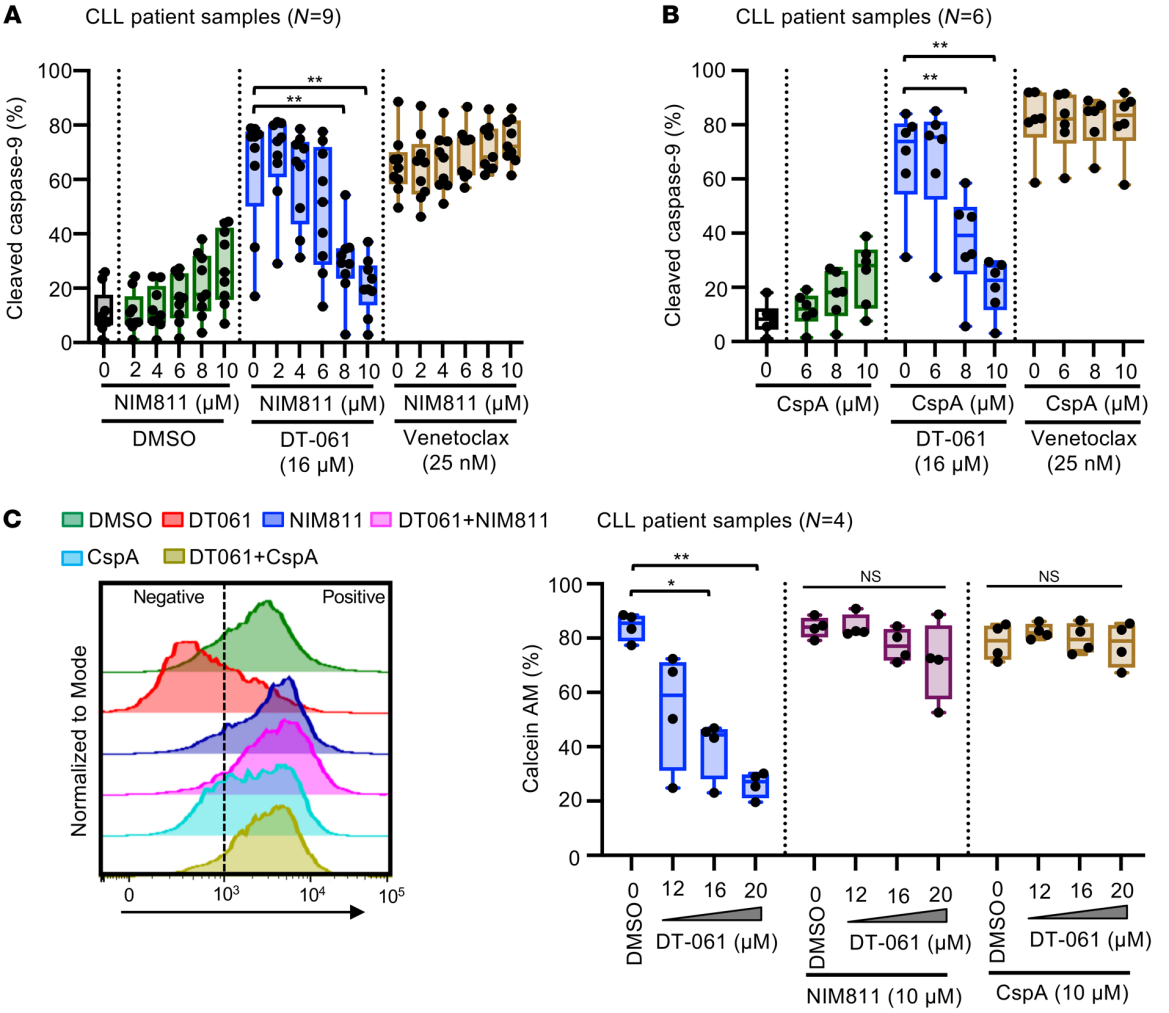
PP2A modulation by DT-061 activates apoptosis in CLL cells by releasing mPTPs. (**A** and **B**) PBMCs from patients with CLL pretreated with increasing concentrations of the CypD inhibitor NIM811 or CspA for 1 hour were incubated with DT-061 (16 μM) or VEN (25 nM) for 12 hours. Apoptosis induction was determined by analyzing cleaved caspase 9, cleaved PARP, and viability dye staining in CLL cells using flow cytometry (cleaved PARP and viability dye data are included in Supplemental Figure 7). Data are presented as box plots showing the percentage of CLL (CD5^+^CD19^+^) cells positive for cleaved caspase-9. (**C**) Samples from patients with CLL pretreated with the CypD inhibitor NIM811 (10 μM) or CspA (10 μM) were incubated with DT-061 (12, 16, and 20 μM) for 6 hours, and mPTP opening in CLL (CD5^+^CD19^+^) cells was assessed using flow cytometry as described in Methods. Stacked histograms show calcein AM staining in CLL cells subjected to various treatments (left panel). Box plots show the percentage CLL cells positive for Calcein AM staining in multiple patient samples treated with DT-061 with or without NIM811 or CspA pretreatment (right panel). Statistical significance was determined by ANOVA with Šidák’s post hoc test for multiple comparisons. *P < 0.05 and **P < 0.01. Data are presented as the mean ± SD.

The underlying mechanism for Bax/Bak-independent apoptosis induction by DT-061 has yet to be resolved. We explored this by systematically examining the cellular processes known to regulate Bax/Bak-independent cytotoxicity such as GSK3β signaling, calcium signaling, oxidative stress, and mPTP induction (45–50). Our results demonstrated that only the inhibitors of cyclophilin D (NIM811 and cyclosporin A [CspA]) that are known to mediate mPTP induction in the mitochondria were able to block the cytotoxicity induced by DT-061 in both WT as well as Bax/Bak-DKO MEC1 CLL cells (Supplemental Figure 6). Subsequently, using flow cytometric analysis of cleaved caspase-9, cleaved PARP, or viability dye staining as described in Figure 5, A and B, we examined apoptosis induction by DT-061 in primary CLL cells pretreated with NIM811 or CspA. Strikingly, our results demonstrated a significant reduction in apoptosis induction by DT-061 in NIM811- as well as CspA-pretreated primary CLL cells (Figure 5, A and B, Supplemental Figure 7, A and C). As expected, viability dye staining was also significantly decreased in NIM811- or CspA-pretreated primary CLL cells (Supplemental Figure 7, B and D). We obtained similar results in Bax/Bak-DKO MEC1 cells (Supplemental Figure 7, E–H), consistent with our data in Supplemental Figure 6. As expected, apoptosis induction by the Bcl-2 inhibitor VEN, which functions through the Bax/Bax pathway, was not affected by pretreatment with NIM811 or CspA (Figure 5, A and B, and Supplemental Figure 7, A–D). These results suggest that the PP2A activator DT-061 triggers apoptosis in CLL cells through induction of mPTP in the mitochondria. To further confirm this, we assessed mPTP induction in primary CLL cells treated with DT-061 using the calcein acetoxymethyl ester–cobalt chloride (calcein AM–cobalt chloride) quenching method, as described previously (51, 52) and in Methods. Consistent with our above results, the signal intensity for calcein AM was significantly reduced in DT-061–treated CLL cells, which was effectively rescued in cells pretreated with CypD inhibitors (NIM811 and CspA) (Figure 5C). Together, these results suggest that PP2A activation by DT-061 triggered apoptosis in CLL cells through induction of mPTP.

### DT-061 overcomes antiapoptotic multidrug resistance in a CLL xenograft mouse model in vivo.

**Figure 6:**
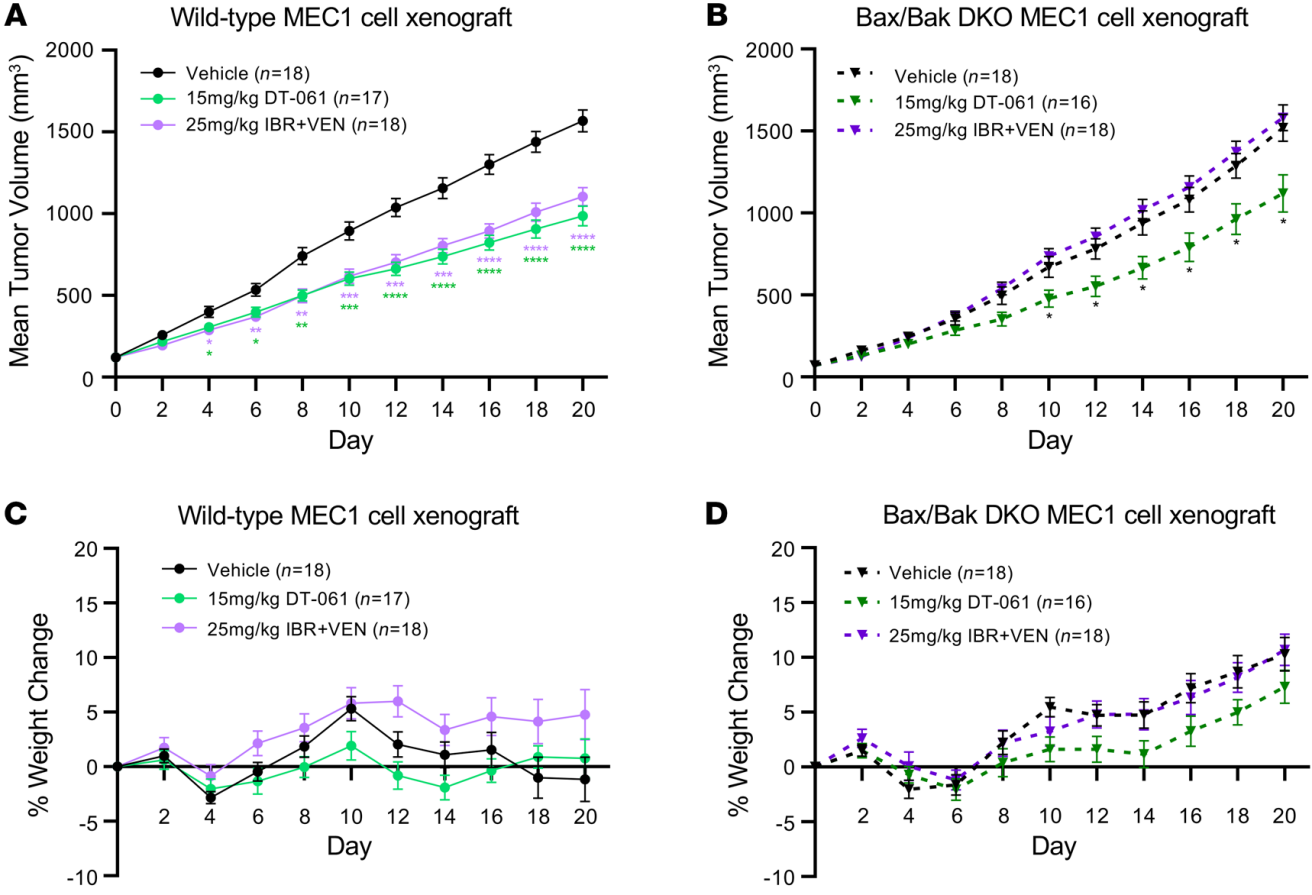
DT-061 overcomes antiapoptotic multidrug resistance in CLL xenograft mouse model in vivo. (**A**) Tumor growth in mice subcutaneously inoculated with the WT MEC1 cell line and treated with vehicle, DT-061, or the combination of IBR and VEN as indicated. (**B**) Tumor growth in mice inoculated with Bax/Bak-DKO MEC1 cells and treated with vehicle, DT-061, or the combination of IBR and VEN. (**C** and **D**) Percentage of body weight change during drug treatment in mice inoculated with WT (**C**) or Bax/Bak-DKO (**D**) MEC1 cells. Data are presented as the mean ± SEM. Statistical significance was determined by 2-way ANOVA with Dunnet’s post hoc test for multiple comparisons. *P < 0.05, **P < 0.01, ***P < 0.001, and ****P < 0.0001.

Last, we sought to demonstrate whether SMAP (DT-061) would inhibit CLL cell growth in vivo. To do so, we utilized the MEC1 cell line in Rag2^−/−^γC^−/−^ mice as previously published (53). To test whether DT-061 has anticancer activity independent of Bax/Bak, as we observed in Figures 4 and 5, we subcutaneously inoculated mice with the WT or Bax/Bak-DKO MEC1 cell line and treated each group with vehicle, 15 mg/kg DT-061, or the combination of IBR (25 mg/kg) and VEN (25 mg/kg). The dosage for DT-061 was selected on the basis of previous in vivo efficacy studies (22–24) and confirmed by us in a dose-finding study in a WT MEC1 xenograft model (Supplemental Figure 8). The dosage for IBR and VEN was selected on the basis of several previously published in vivo studies (25, 54, 55). The combination of IBR and VEN exhibited exceptional anticancer activity in clinical trials in patients with leukemia or lymphoma, including those with CLL (11, 56), and it was recently approved by the FDA for the treatment of MCL. Notably, DT-061 inhibited the growth of both WT (~40.5%) and Bax/Bak-DKO (~27.6%) CLL cells in these mice in vivo (Figure 6, A and B), consistent with our ex vivo data showing that DT-061 inhibited tumor growth independent of the classical Bax/Bak pathway. In contrast to DT-061 treatment, the combination of IBR and VEN inhibited tumor growth only in WT mice (~32.6%), but not the Bax/Bak-DKO group (~–4.3%) (Figure 6, A and B), further suggesting that the in vivo activity of this drug combination was dependent on the Bax/Bak pathway. Interestingly, we noted that DT-061, but not the IBR and VEN combination, significantly suppressed MEC1 cell tumor growth in visceral organs such as liver, spleen, and kidney (Supplemental Figure 9), suggesting that DT-061 was equally effective in suppressing CLL cell growth in the tissue microenvironment in vivo in which drug-resistant leukemic B cells persist during treatment (4). In addition, we observed no significant change in body weights of the mice between the WT and Bax/Bak-DKO treatment groups (Figure 6, C and D). The Bax/Bak-DKO status was confirmed by Western blot (WB) analysis in the xenograft tissues at the end of the study (data not shown). Collectively, our results indicate that DT-061 inhibited tumor growth in a Bax/Bak-independent manner and suggest that PP2A activators could be used therapeutically for the treatment of multidrug-resistant CLL.

## Discussion

We identified CLL cells in vivo that displayed defective activation of Bax/Bak proteins, which resulted in the development of resistance to multiple BH domain antagonists. Based on our co-immunoprecipitation studies in CLL cells treated with BH domain antagonists ex vivo (Figure 2), we suggest that proapoptotic protein swapping by the upregulated antiapoptotic proteins prevented the activation of Bax/Bak proteins in these apoptosis-resistant CLL cells. Supporting this observation, we and others have previously demonstrated the overexpression of multiple antiapoptotic proteins in drug-resistant CLL cells and cells that survive VEN treatment in vivo (4, 15, 18). Although the binding of Bim to Mcl-1 was decreased in cells treated with S63845 (Mcl-1 inhibitor), only a small fraction of Bim shifted to Bcl-xL (Figure 2), suggesting either the involvement of additional antiapoptotic proteins or alternative resistance mechanisms. Consistent with these observations, other groups have reported the genetic/epigenetic-driven impairment of Bax/Bak proteins (57, 58), which would also be expected to generate this antiapoptotic, multidrug-resistant phenotype.

The presence of cancer cells displaying apoptosis resistance due to insufficient activation of Bax/Bak in vivo presents a formidable therapeutic challenge, as a majority of cancer drugs rely on Bax/Bak-dependent apoptosis (44). Underscoring this, studies have linked altered Bax/Bak expression and function to worse clinical outcomes in B cell malignancies and other cancers (59–62). We and others have demonstrated that a combination of antiapoptotic protein inhibitors is able to overcome multidrug resistance in CLL cells ex vivo. However, this approach is likely to generate significant off-target toxicities in patients, as suggested in several reports (63, 64). Thus, alternative treatments with novel mechanisms of activation are needed to deplete these populations of multidrug-resistant cancer cells in vivo.

The induction of Bax/Bak-dependent intrinsic apoptosis is the primary driver of the activity of many cancer therapies. Emerging data clearly suggest that defects in Bax/Bak pathway components decrease cancer cell sensitivity to proapoptotic therapies, including IBR and VEN. Thus, novel agents that activate Bax/Bak-independent programmed cell death mechanisms (i.e., ferroptosis, mitoptosis, pyroptosis, etc.) are gaining increasing interest as alternative cancer treatments (65). Our data demonstrate that the SMAP (DT-061) overcame multidrug resistance in CLL cells through the activation of Bax/Bak-independent apoptosis, which was triggered by the induction of mPTPs (Figures 4–6, and Supplemental Figures 5–7). The ability of SMAPs to induce apoptosis independent of Bax/Bak offers a unique therapeutic opportunity in the relapsed/refractory and treatment-resistant settings. While we demonstrate that PP2A activation by DT-061 led to Bax/Bak-independent apoptosis, other groups have suggested that PP2A also plays a role in Bax/Bak-dependent apoptosis (66). In this study, we noticed that Bax/Bak-DKO cells were slightly less sensitive to DT-061 than were WT cells (Figure 6 and Supplemental Figure 5A), underscoring that PP2A may also play a role in Bax/Bak-dependent apoptosis. While the majority of our data clearly suggest that DT-061 functions primarily through mPTP-dependent apoptosis in CLL, the potential involvement of other cell death pathways in different cancer models and contexts cannot be ruled out.

The mPTP is a putative pore-like structure responsible for the induction of mitochondrial permeability transition and cell death (50, 67). Although the mPTP structure is not fully resolved, studies have implicated various proteins including voltage-dependent anion channels (VDACs), adenosine nucleotide transporter (ANT), and CypD in pore formation in mitochondria, with CypD being the most studied and its inhibition having been consistently demonstrated to block mPTP induction (67). There are 17 different cyclophilin proteins expressed in human cells, and only CypD is associated with mPTP induction in the mitochondria. In our analysis, pretreatment with 2 known CypD inhibitors (NIM811 and CspA) effectively blocked the induction of apoptosis as well as the release of calcein AM from the mitochondria in DT-061–treated CLL cells (Figure 5 and Supplemental Figure 7), suggesting that PP2A activation overcame multidrug resistance through mPTP induction–dependent apoptosis.

Various cellular processes including oxidative stress, calcium signaling, and GSK3β signaling have been implicated in mPTP induction in human cells (45–50). While PP2A has been shown to regulate some of these cellular processes, our data failed to demonstrate any involvement of these processes in DT-061–induced cytotoxicity (Supplemental Figure 6). Pagano et al. demonstrated that disruption of the PP2A-SET complex using an alkoxy phenyl-1-propanone derivative induces apoptosis in CLL cells as a result of Bad dephosphorylation and/or upregulation of Bim (66). Another study reported that the PP2A-dependent dephosphorylation of BAD induces mPTP induction in cells, although the underlying mechanism is not clear (68). However, we detected no change in the expression of Bim or Bad proteins in DT-061–treated CLL cells (data not shown), potentially because of differences in cell type and/or the PP2A activation mechanism used in our study. How SMAP-driven PP2A activation triggers mPTP opening in CLL cells is a fruitful area for future investigation.

In summary, we demonstrate the presence of leukemic B cells displaying pre-mitochondrial apoptosis restriction due to defective activation of Bax/Bak in patients with CLL de novo. These cells exhibited enrichment in patients undergoing treatment with the proapoptotic agent VEN. SMAPs (DT-061) overcame this restriction through induction of Bax/Bak-independent apoptosis, which was mediated by the induction of mPTPs. Collectively, our work presents a pharmaceutically tractable pathway to deplete this drug-resistant cancer cell reservoir.

## Methods

### Patient sample preparation and analysis.

The blood samples were processed into PBMCs by Ficoll density-gradient centrifugation at the Biorepository and Tissue Research Facility (BTRF) of the UVA. PBMC samples, freshly frozen in liquid nitrogen with 90% FCS and 10% DMSO, were used. Patient samples were cultured in HEPES/pyruvate-supplemented RPMI containing 10% FCS. For ex vivo drug screening of patient samples (Figure 3, A and B, Figure 4A, and Supplemental Figure 1), patient PBMC samples were cultured with or without agonists (CpG-ODN [1.5 μg/mL] + sCD40L [2 μg/mL] + IL-10 [15 ng/mL]; “agonist mix”) for 12 hours and treated with various inhibitors as well as a second dose of agonist mix as described previously (15) and in the figure legends. At the end of the drug treatment, cells were subjected to downstream analysis as indicated. Details on the antibodies, drugs, reagents, and patients’ characteristics are provided in Supplemental Tables 1–3.

### ATA.

Bax and Bak activation in patient samples was assessed using a modification of the established BH3-profiling assay (69–71), which we termed the “apoptosis threshold assay” (ATA). We assessed Bax/Bak activation dependency on Bcl-2, Mcl-1, or Bcl-xL using the BH3 mimetics VEN (Bcl-2 inhibitor) (72), S63845 (Mcl-1 inhibitor) (73), or A1155463 (Bcl-xL inhibitor) (74), respectively. Freshly thawed patient PBMCs were stained with Live/Dead near-infrared viability dye and for surface markers using anti–CD5-APC, anti–CD19-BV421, and anti–CD69-BV605 for 20 minutes at 37°C. PBMCs were washed and incubated with BH3 mimetics in RPMI containing 10% FCS at 37°C for 3 hours. Subsequently, cells were fixed in paraformaldehyde (1.6%), permeabilized using saponin, and stained with anti–Bax-PE (Bax clone 6A7), anti–Bak-PE/Cy7 (Bak clone G317-2), or anti–cleaved caspase-9 followed by anti–rabbit AF488 antibody. The anti-Bax (clone 6A7) (41–43) and anti-Bak (clone G317-2) recognize active confirmation of the Bax and Bak proteins, respectively. Anti-Bax (clone 6A7) and anti-Bak (clone G317-2) were conjugated with PE and PE/Cy7, respectively, using Lightning-Link conjugation kits (Abcam), as per the manufacturer’s instructions. Antibody details are provided in Supplemental Table 1.

### IP and protein interaction analysis to determine proapoptotic protein Bim swapping between antiapoptotic proteins.

IP was performed as described earlier (75). Briefly, patient PBMCs containing more than 90% CLL cells were preincubated with the agonist mix for 12 hours as described above to induce multidrug resistance (see Supplemental Figure 1). Then, cells were treated with antiapoptotic protein inhibitors for 12 hours along with a second dose of the agonist mix. Samples were lysed on ice for 30 minutes using 0.2% NP40 lysis buffer containing a protease inhibitor cocktail, and lysates were precleared by incubating with protein A/G–coated magnetic beads (Pierce Biotechnology, Thermo Fisher Scientific) for 2 hours at 4°C with continuous shaking. Then, lysates were incubated with anti-Bim antibody (clone C34C5) for 12 hours at 4°C and immunoprecipitated using protein A/G–coated magnetic beads. The presence of Bim, Mcl-1, Bcl-xL, and Bcl-2 proteins in IP samples was analyzed by Western blotting. Details on the reagents and antibodies used are provided in Supplemental Table 1.

### Flow cytometric analysis of mPTPs.

mPTP opening in CLL cells was assessed using the MitoProbe Transition Pore Assay Kit (Molecular Probes), as described earlier (51, 52). Briefly, patient PBMCs were stained with Live/Dead near-infrared viability stain, anti–CD5-APC, and anti–CD19-BV421 in complete media at 37°C for 20 minutes. Cells were washed with Hank’s buffered salt solution and treated with calcein AM (300 nM) for 15 minutes at 37°C. Cells were treated with 0.4 mM CoCl_2_ with or without NIM811 (10 μM) or CspA (10 μM) for 30 minutes at 37°C. Subsequently, cells were incubated with DT-061 for 6 hours at 37°C. Calcein AM staining was then analyzed by flow cytometry. The opening of mPTPs leads to the release of calcein AM from the mitochondria, allowing calcein AM fluorescence quenching by CoCl_2_ in the cytoplasm. This results in loss of calcein AM staining.

### DT-061 tumor inhibition analysis in vivo using a CLL xenograft mouse model.

MEC1 control (WT) or Bax/Bak-DKO cells (5.0 × 10^6^) were injected subcutaneously into the right flank of Rag2^−/−^γC^−/−^ mice. When tumors reached an average size of 100 mm^3^, mice were treated with vehicle or 15 mg/kg DT-061 twice a day by oral gavage or with 25 mg/kg VEN and IBR 5 days per week as reported previously (25, 54, 55). The tumor sizes were measured by caliper, and body weights were recorded every other day and during treatment as indicated. At study termination, the mice received a final treatment 4 hours before sacrifice. DT-061, VEN, and IBR were delivered by oral gavage in a solution of 10% *N*,*N*-dimethylacetamide (DMA) and 10% Solutol HS 15 (Kolliphor HS 15, MilliporeSigma) in sterile water.

### Statistics.

Results are presented as the mean ± SD, unless indicated otherwise. Statistical significance was determined by 1-way ANOVA or 2-tailed Student’s *t* test, unless specified otherwise, using GraphPad Prism software, and a *P* value of less than 0.05 was considered statistically significant.

### Study approval.

Informed consent was obtained from all patients with CLL upon local IRB approval and in accordance with the Declaration of Helsinki. The murine study was performed according to the University of Michigan’s IACUC-approved protocol PRO00008489.

## Author contributions

KDJ designed and helped perform and analyze most of the experiments and took the lead in writing the manuscript and addressing the reviewers’ comments. SS assisted with Bax/Bak-DKO cell line development, validation, and experimental design. BT, CM, VLG, CCF, CMO, and KPZ performed experiments and helped with data analysis. KMI organized the patients’ histories and interpreted the clinical association. CAP and MEW oversaw the clinical integration and helped with experimental design and interpretation. TPB provided advice on B cell biology and flow cytometric analysis. The overall project was organized and overseen by MK, MJW, and GN.

## Supplementary Material

Supplemental data

Supporting data values

## Version 1 05/11/2023

In-Press Preview

## Version 2 07/03/2023

Electronic publication
