# Supplementary material for: PP2A modulation overcomes multidrug resistance in chronic lymphocytic leukemia via mPTP-dependent apoptosis
Source: J Clin Invest. 2023 Jul 3;133(13):e155938. doi: 10.1172/JCI155938 (PMC10313372; doi:10.1172/JCI155938)
Supplement: Supplemental data [file jci-133-155938-s164.pdf]

Figure S1

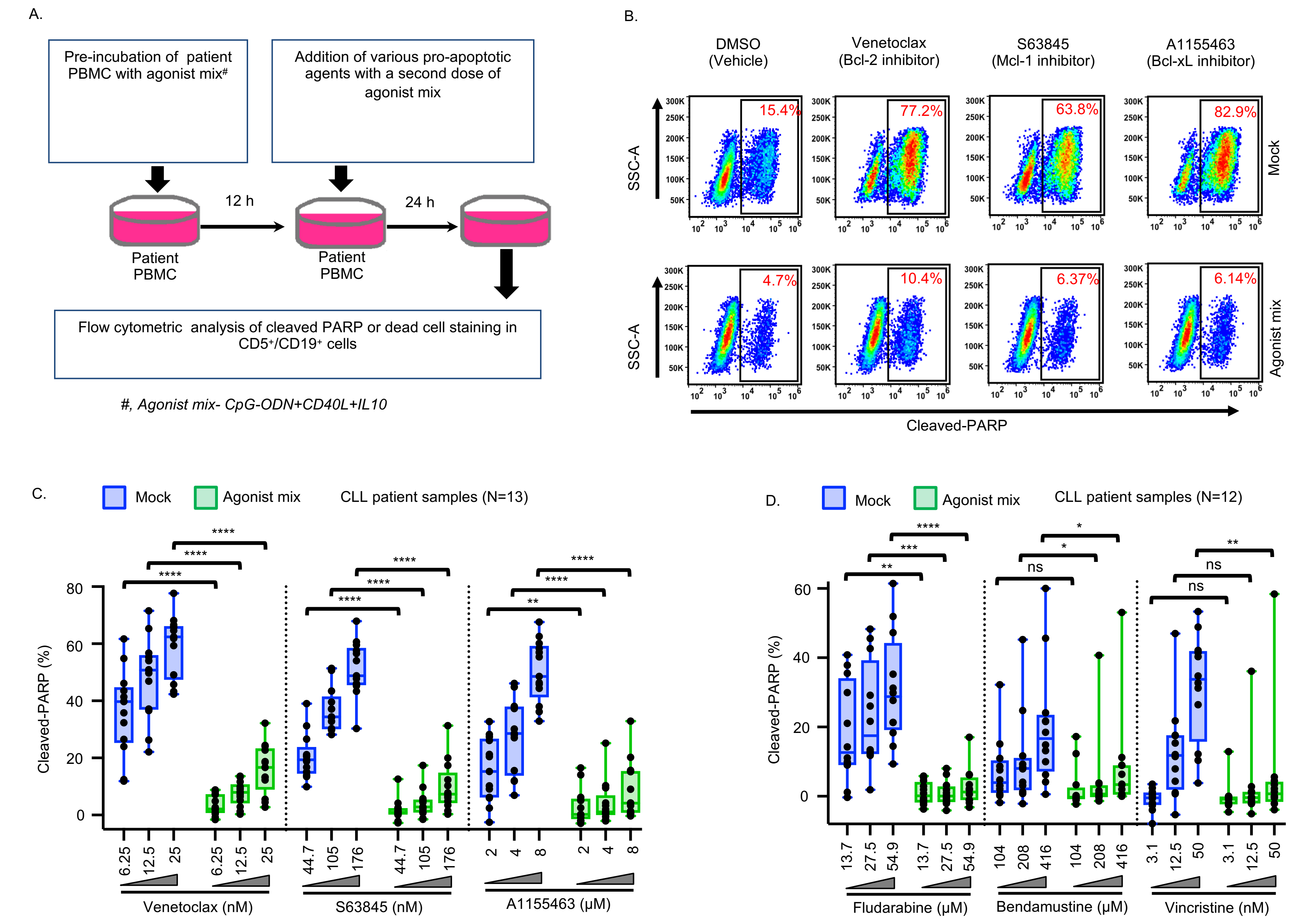

A.
Pre-incubation of patient PBMC with agonist mix#
Addition of various pro-apoptotic agents with a second dose of agonist mix
12 h
24 h
Patient PBMC
Patient PBMC
Flow cytometric analysis of cleaved PARP or dead cell staining in CD5+/CD19+ cells
#, Agonist mix- CpG-ODN+CD40L+IL10
B.
DMSO (Vehicle)
Venetoclax (Bcl-2 inhibitor)
S63845 (Mcl-1 inhibitor)
A1155463 (Bcl-xL inhibitor)
15.4%
77.2%
63.8%
82.9%
Mock
4.7%
10.4%
6.37%
6.14%
Agonist mix
SSC-A
Cleaved-PARP
C.
Mock
Agonist mix
CLL patient samples (N=13)
Cleaved-PARP (%)
Venetoclax (nM)
S63845 (nM)
A1155463 (μM)
D.
Mock
Agonist mix
CLL patient samples (N=12)
Cleaved-PARP (%)
Fludarabine (μM)
Bendamustine (μM)
Vincristine (nM)

Figure S1

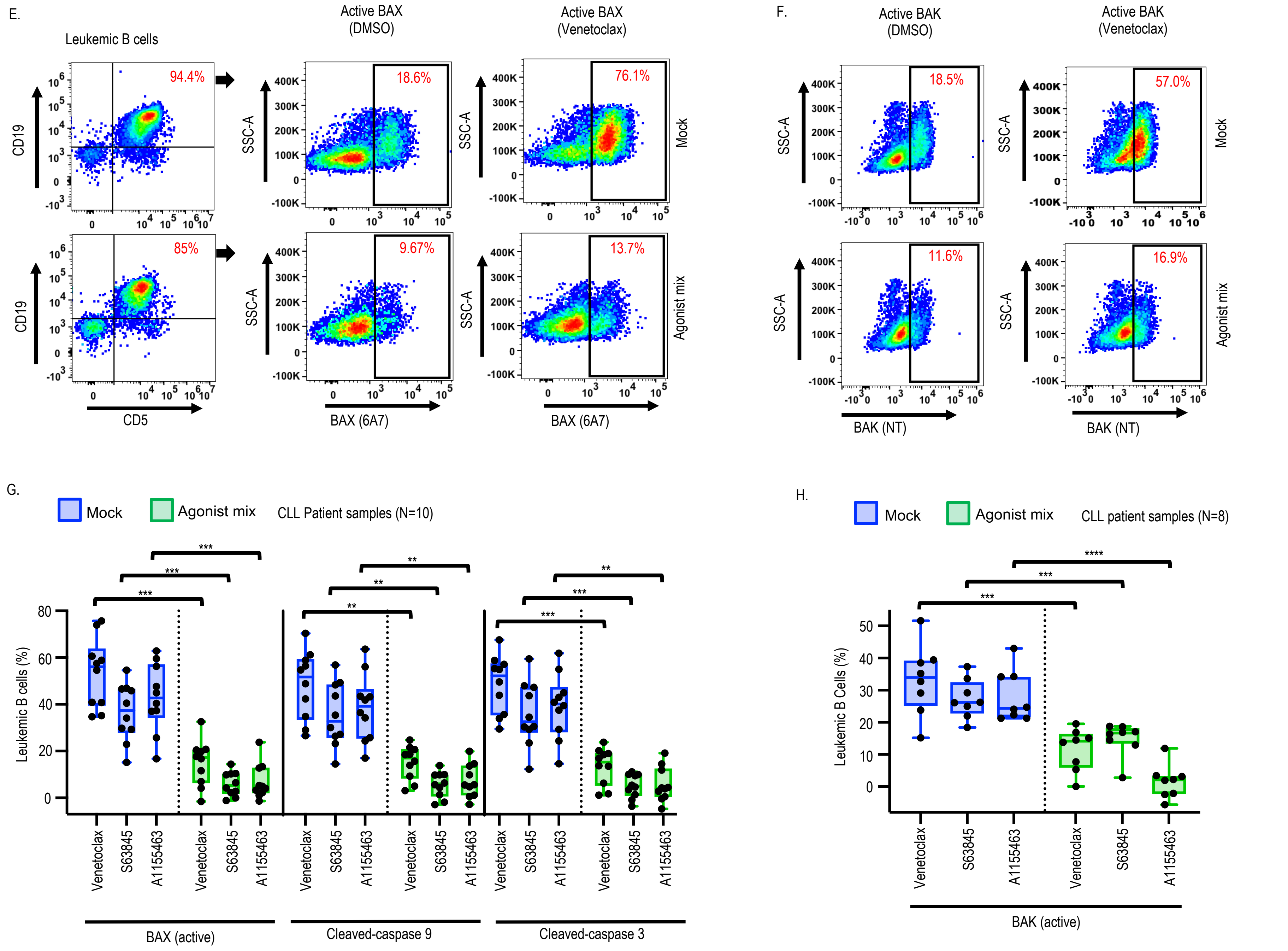

E.
Leukemic B cells
Active BAX (DMSO)
Active BAX (Venetoclax)
94.4%
85%
18.6%
9.67%
76.1%
13.7%
CD19
CD5
SSC-A
BAX (6A7)
Mock
Agonist mix
F.
Active BAK (DMSO)
Active BAK (Venetoclax)
18.5%
57.0%
11.6%
16.9%
BAK (NT)
G.
Mock
Agonist mix
CLL Patient samples (N=10)
Leukemic B cells (%)
Venetoclax
S63845
A1155463
BAX (active)
Cleaved-caspase 9
Cleaved-caspase 3
H.
CLL patient samples (N=8)
Leukemic B Cells (%)
BAK (active)

Figure S2

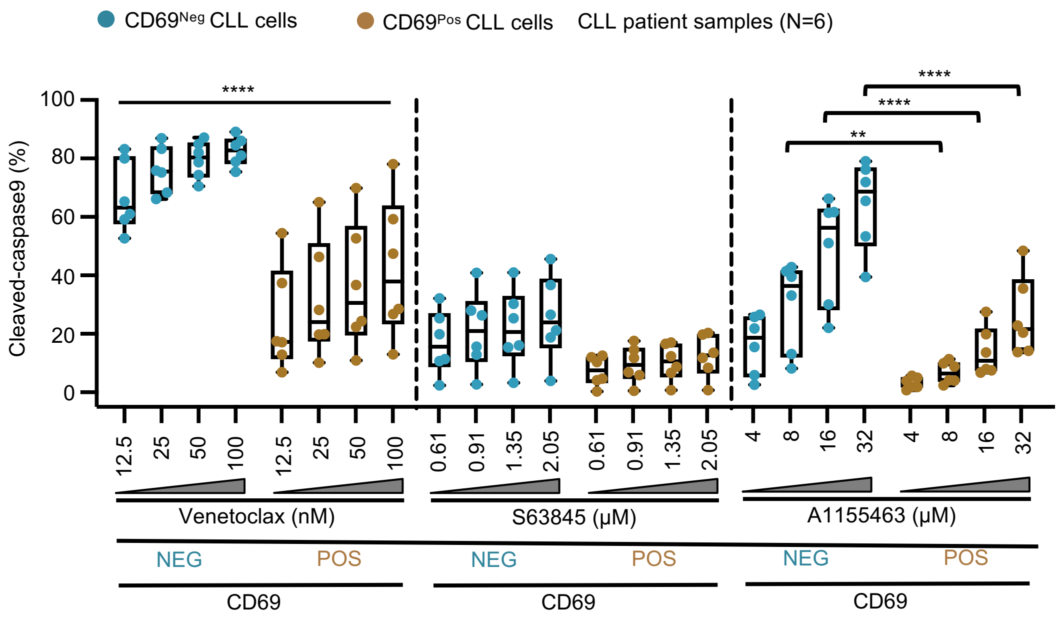

CD69^Neg CLL cells
CD69^Pos CLL cells
CLL patient samples (N=6)
Cleaved-caspase9 (%)
100
80
60
40
20
0
****
**
****
****
12.5
25
50
100
12.5
25
50
100
0.61
0.91
1.35
2.05
0.61
0.91
1.35
2.05
4
8
16
32
4
8
16
32
Venetoclax (nM)
S63845 (μM)
A1155463 (μM)
NEG
POS
NEG
POS
NEG
POS
CD69
CD69
CD69

Figure S3

A.

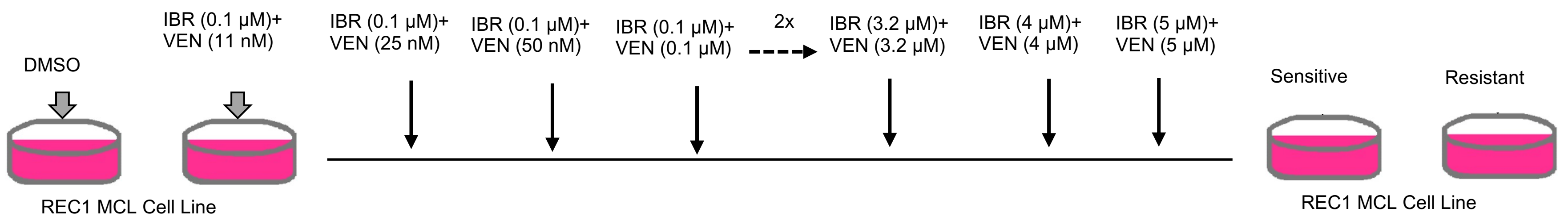
DMSO
IBR (0.1 µM)+
VEN (11 nM)
IBR (0.1 µM)+
VEN (25 nM)
IBR (0.1 µM)+
VEN (50 nM)
IBR (0.1 µM)+
VEN (0.1 µM)
2x
IBR (3.2 µM)+
VEN (3.2 µM)
IBR (4 µM)+
VEN (4 µM)
IBR (5 µM)+
VEN (5 µM)
Sensitive
Resistant
REC1 MCL Cell Line
REC1 MCL Cell Line

B.

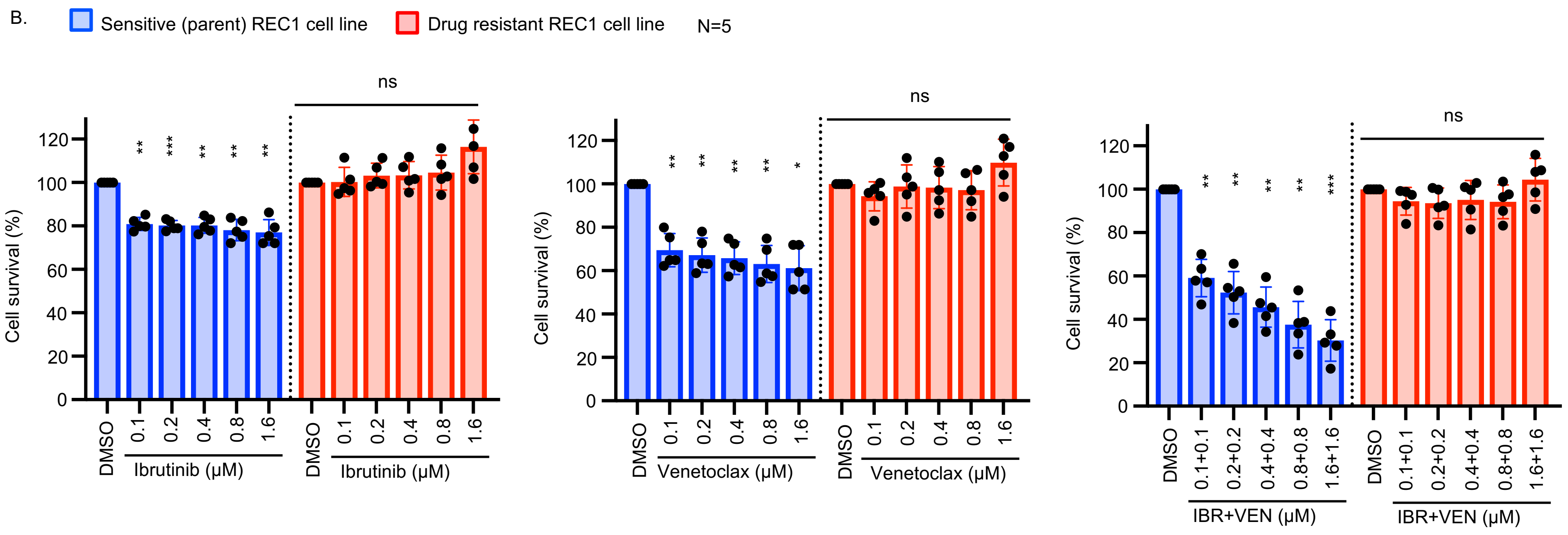
Sensitive (parent) REC1 cell line
Drug resistant REC1 cell line
N=5
ns
Cell survival (%)
Ibrutinib (µM)
Venetoclax (µM)
IBR+VEN (µM)
DMSO
0.1
0.2
0.4
0.8
1.6
0.1+0.1
0.2+0.2
0.4+0.4
0.8+0.8
1.6+1.6

Figure S3

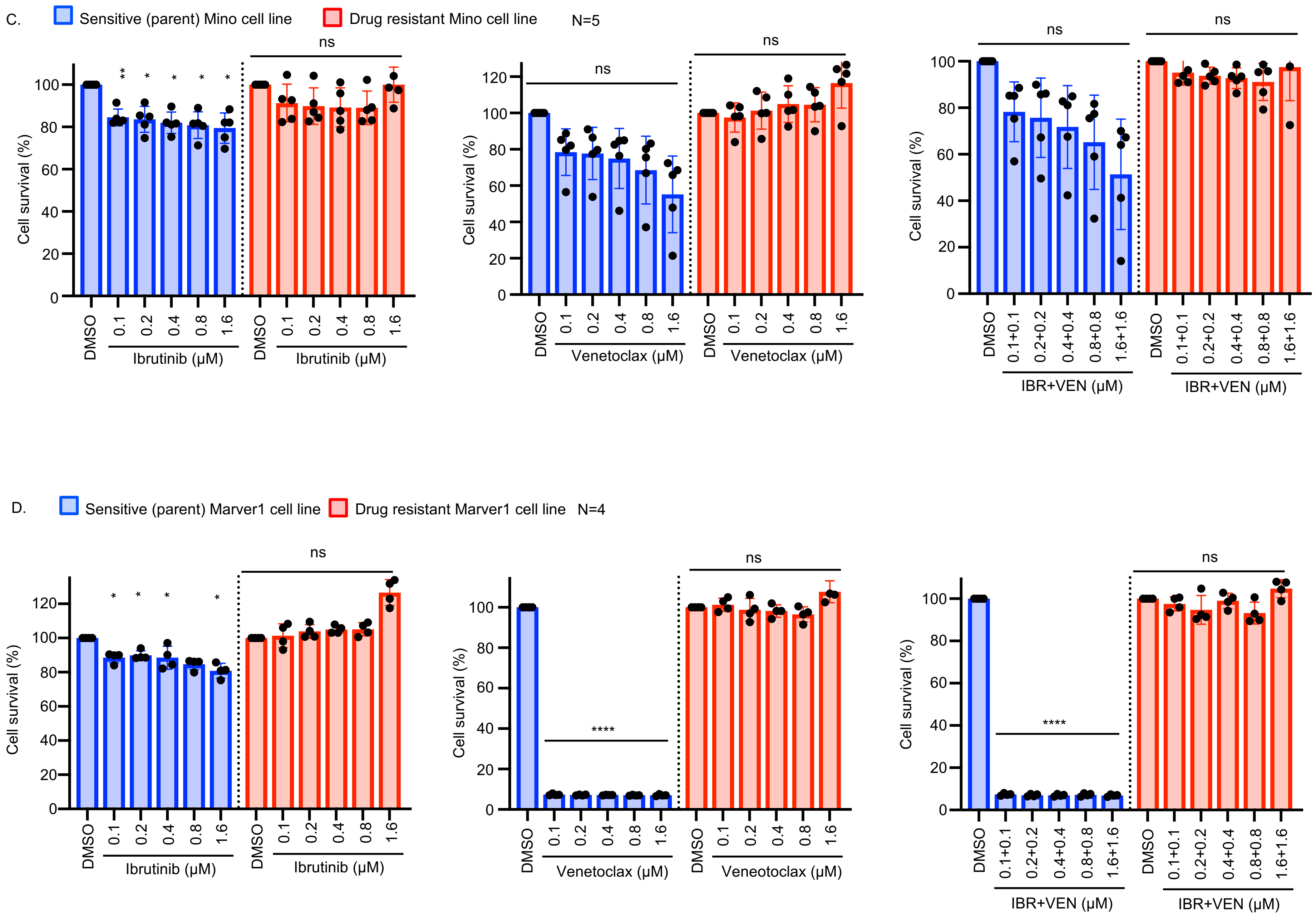
C.
Sensitive (parent) Mino cell line
Drug resistant Mino cell line
N=5
Cell survival (%)
0
20
40
60
80
100
120
ns
**
*
DMSO
0.1
0.2
0.4
0.8
1.6
Ibrutinib (μM)
Venetoclax (μM)
0.1+0.1
0.2+0.2
0.4+0.4
0.8+0.8
1.6+1.6
IBR+VEN (μM)
D.
Sensitive (parent) Marver1 cell line
Drug resistant Marver1 cell line
N=4
****
Veneotoclax (μM)

Figure S4

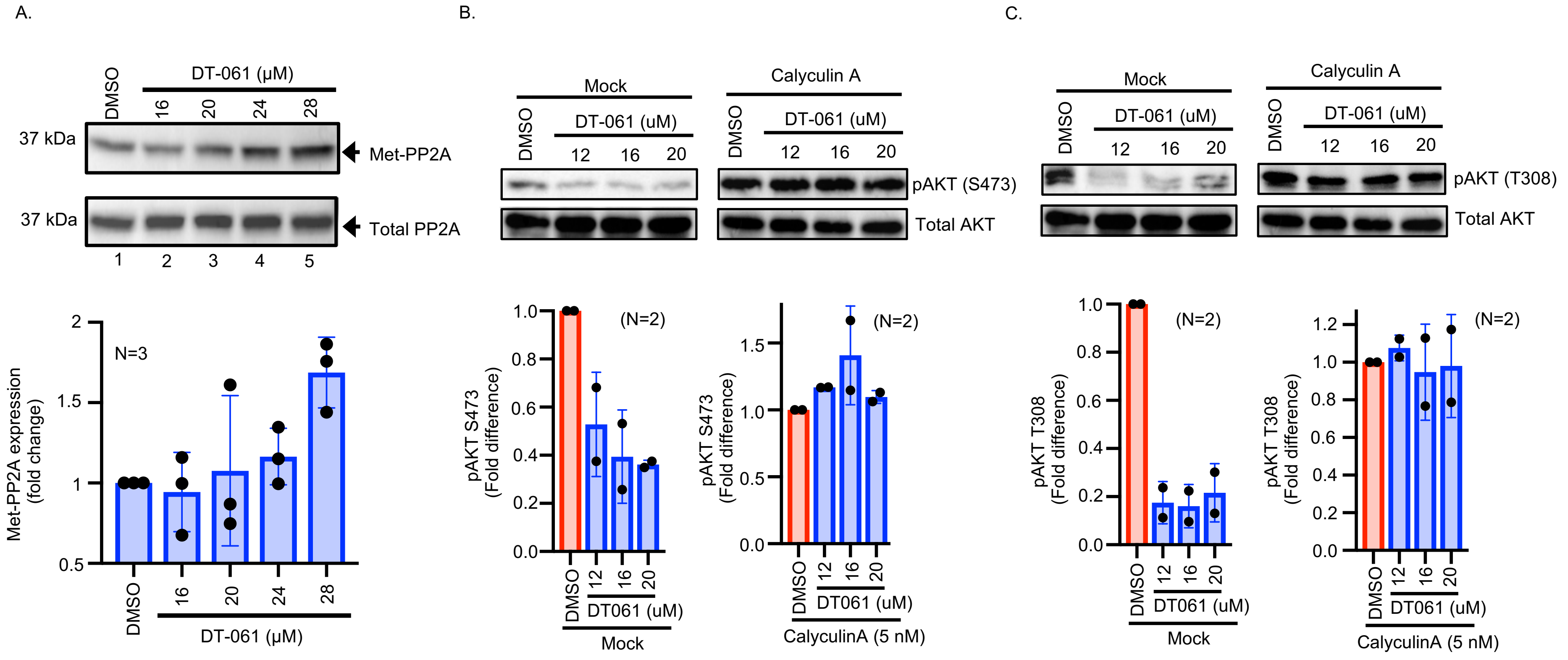

A.
DMSO
DT-061 (μM)
16
20
24
28
37 kDa
Met-PP2A
37 kDa
Total PP2A
1
2
3
4
5
Met-PP2A expression
(fold change)
N=3
2
1.5
1
0.5
DMSO
16
20
24
28
DT-061 (μM)
B.
Mock
DMSO
DT-061 (uM)
12
16
20
Calyculin A
DMSO
DT-061 (uM)
12
16
20
pAKT (S473)
Total AKT
pAKT S473
(Fold difference)
1.0
0.8
0.6
0.4
0.2
0.0
(N=2)
DMSO
12
16
20
DT061 (uM)
Mock
pAKT S473
(Fold difference)
1.5
1.0
0.5
0.0
(N=2)
DMSO
12
16
20
DT061 (uM)
CalyculinA (5 nM)
C.
Mock
DMSO
DT-061 (uM)
12
16
20
Calyculin A
DMSO
DT-061 (uM)
12
16
20
pAKT (T308)
Total AKT
pAKT T308
(Fold difference)
1.0
0.8
0.6
0.4
0.2
0.0
(N=2)
DMSO
12
16
20
DT061 (uM)
Mock
pAKT T308
(Fold difference)
1.2
1.0
0.8
0.6
0.4
0.2
0.0
(N=2)
DMSO
12
16
20
DT061 (uM)
CalyculinA (5 nM)

Figure S5

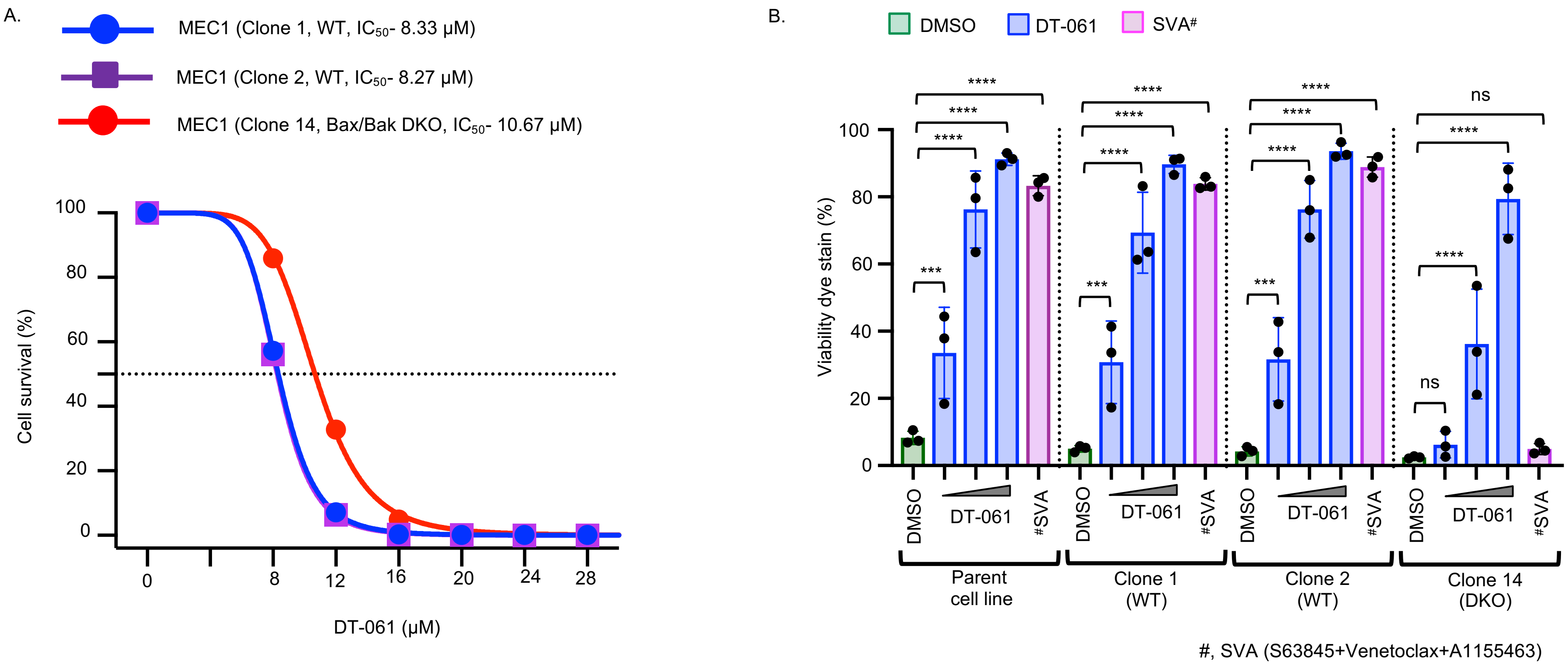
A.
MEC1 (Clone 1, WT, IC50- 8.33 μM)
MEC1 (Clone 2, WT, IC50- 8.27 μM)
MEC1 (Clone 14, Bax/Bak DKO, IC50- 10.67 μM)
Cell survival (%)
100
80
60
40
20
0
0
8
12
16
20
24
28
DT-061 (μM)
B.
DMSO
DT-061
SVA#
Viability dye stain (%)
100
80
60
40
20
0
***
****
ns
DMSO
DT-061
#SVA
Parent cell line
Clone 1 (WT)
Clone 2 (WT)
Clone 14 (DKO)
#, SVA (S63845+Venetoclax+A1155463)

Figure S6

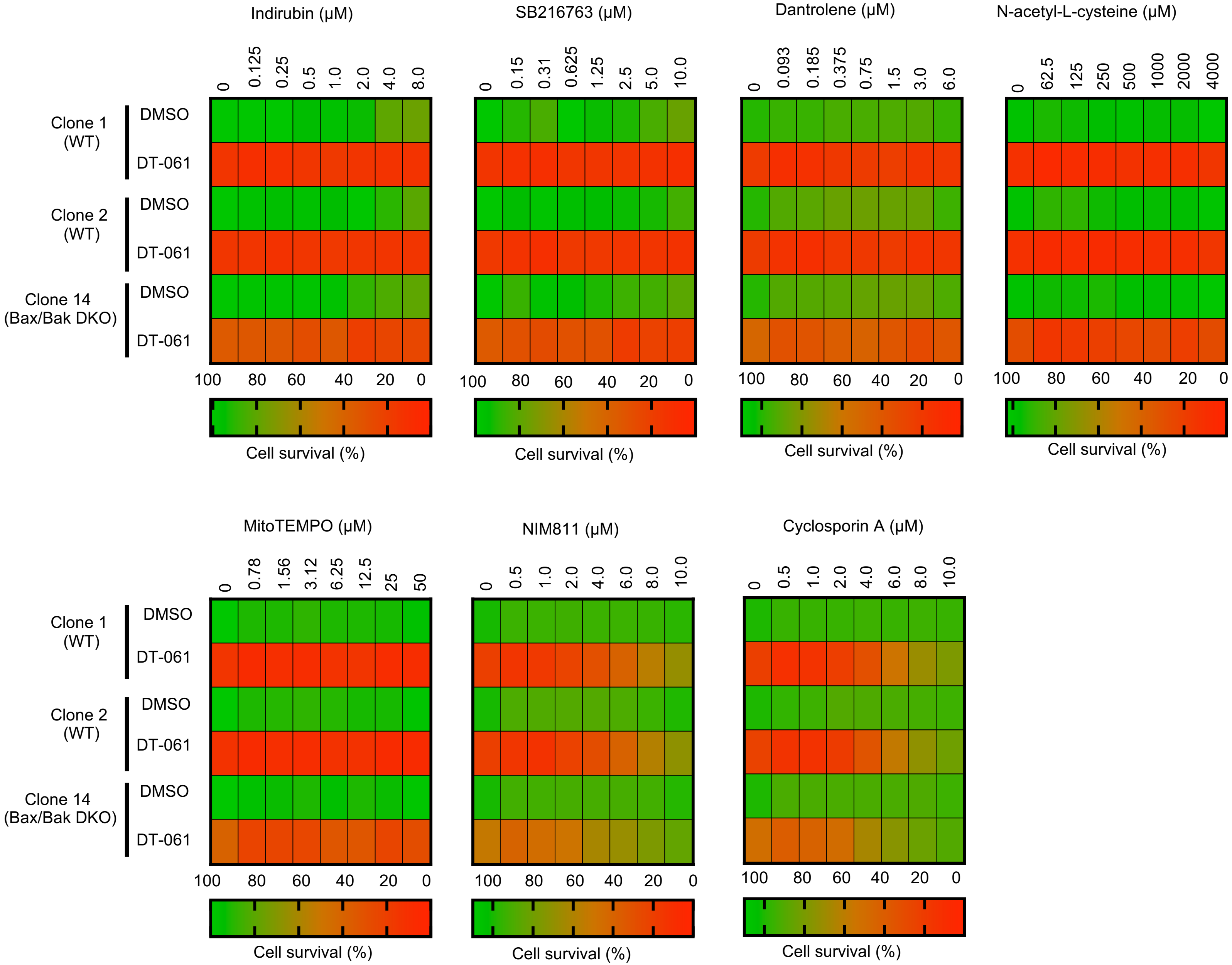
Indirubin (μM)
0 0.125 0.25 0.5 1.0 2.0 4.0 8.0
SB216763 (μM)
0 0.15 0.31 0.625 1.25 2.5 5.0 10.0
Dantrolene (μM)
0 0.093 0.185 0.375 0.75 1.5 3.0 6.0
N-acetyl-L-cysteine (μM)
0 62.5 125 250 500 1000 2000 4000
MitoTEMPO (μM)
0 0.78 1.56 3.12 6.25 12.5 25 50
NIM811 (μM)
0 0.5 1.0 2.0 4.0 6.0 8.0 10.0
Cyclosporin A (μM)
0 0.5 1.0 2.0 4.0 6.0 8.0 10.0
Clone 1 (WT)
Clone 2 (WT)
Clone 14 (Bax/Bak DKO)
DMSO
DT-061
100 80 60 40 20 0
Cell survival (%)

Figure S7

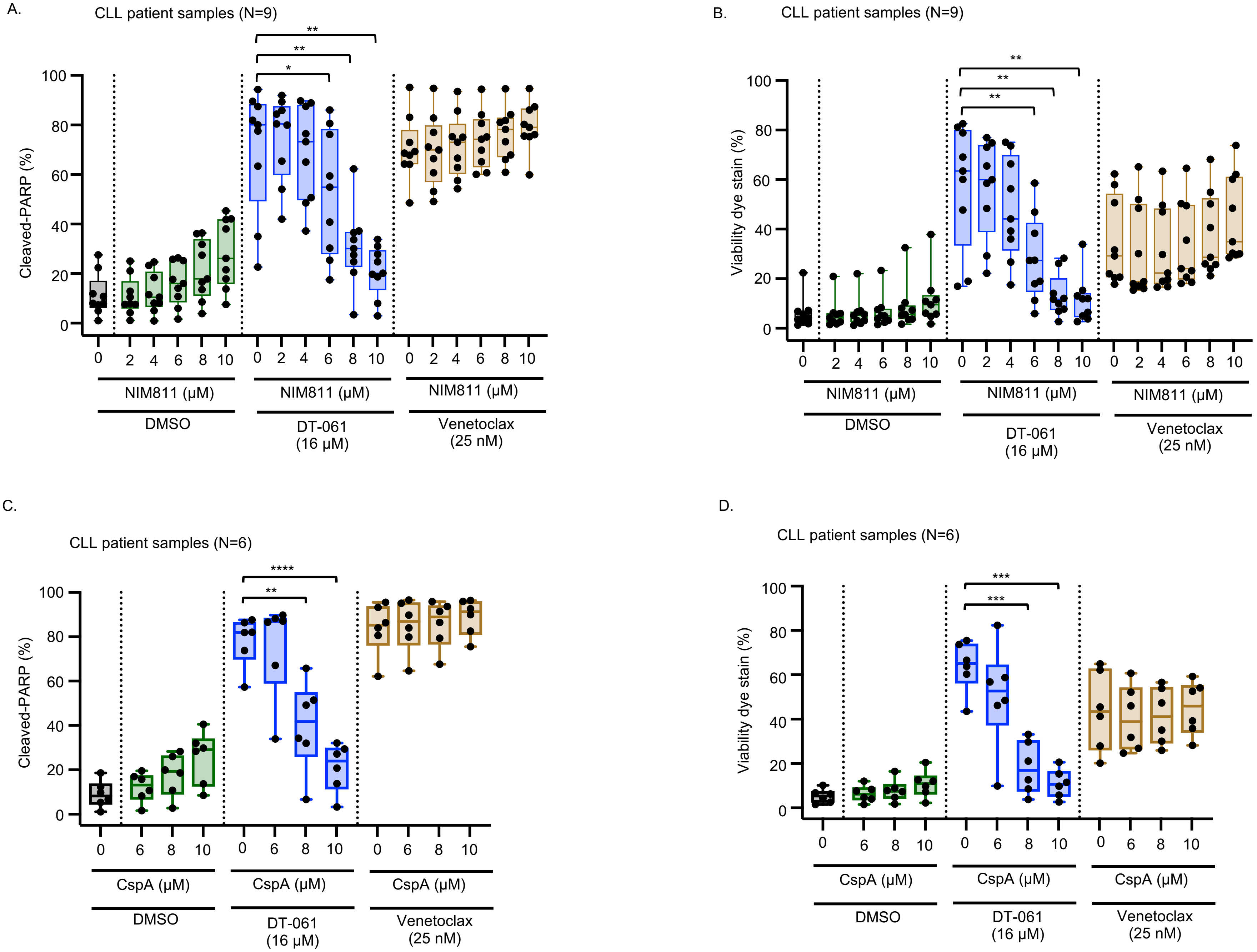
A.
CLL patient samples (N=9)
Cleaved-PARP (%)
0
20
40
60
80
100
*
**
**
0
2
4
6
8
10
0
2
4
6
8
10
0
2
4
6
8
10
NIM811 (μM)
NIM811 (μM)
NIM811 (μM)
DMSO
DT-061
(16 μM)
Venetoclax
(25 nM)
B.
CLL patient samples (N=9)
Viability dye stain (%)
0
20
40
60
80
100
**
**
**
0
2
4
6
8
10
0
2
4
6
8
10
0
2
4
6
8
10
NIM811 (μM)
NIM811 (μM)
NIM811 (μM)
DMSO
DT-061
(16 μM)
Venetoclax
(25 nM)
C.
CLL patient samples (N=6)
Cleaved-PARP (%)
0
20
40
60
80
100
**
****
0
6
8
10
0
6
8
10
0
6
8
10
CspA (μM)
CspA (μM)
CspA (μM)
DMSO
DT-061
(16 μM)
Venetoclax
(25 nM)
D.
CLL patient samples (N=6)
Viability dye stain (%)
0
20
40
60
80
100
***
***
0
6
8
10
0
6
8
10
0
6
8
10
CspA (μM)
CspA (μM)
CspA (μM)
DMSO
DT-061
(16 μM)
Venetoclax
(25 nM)

Figure S7

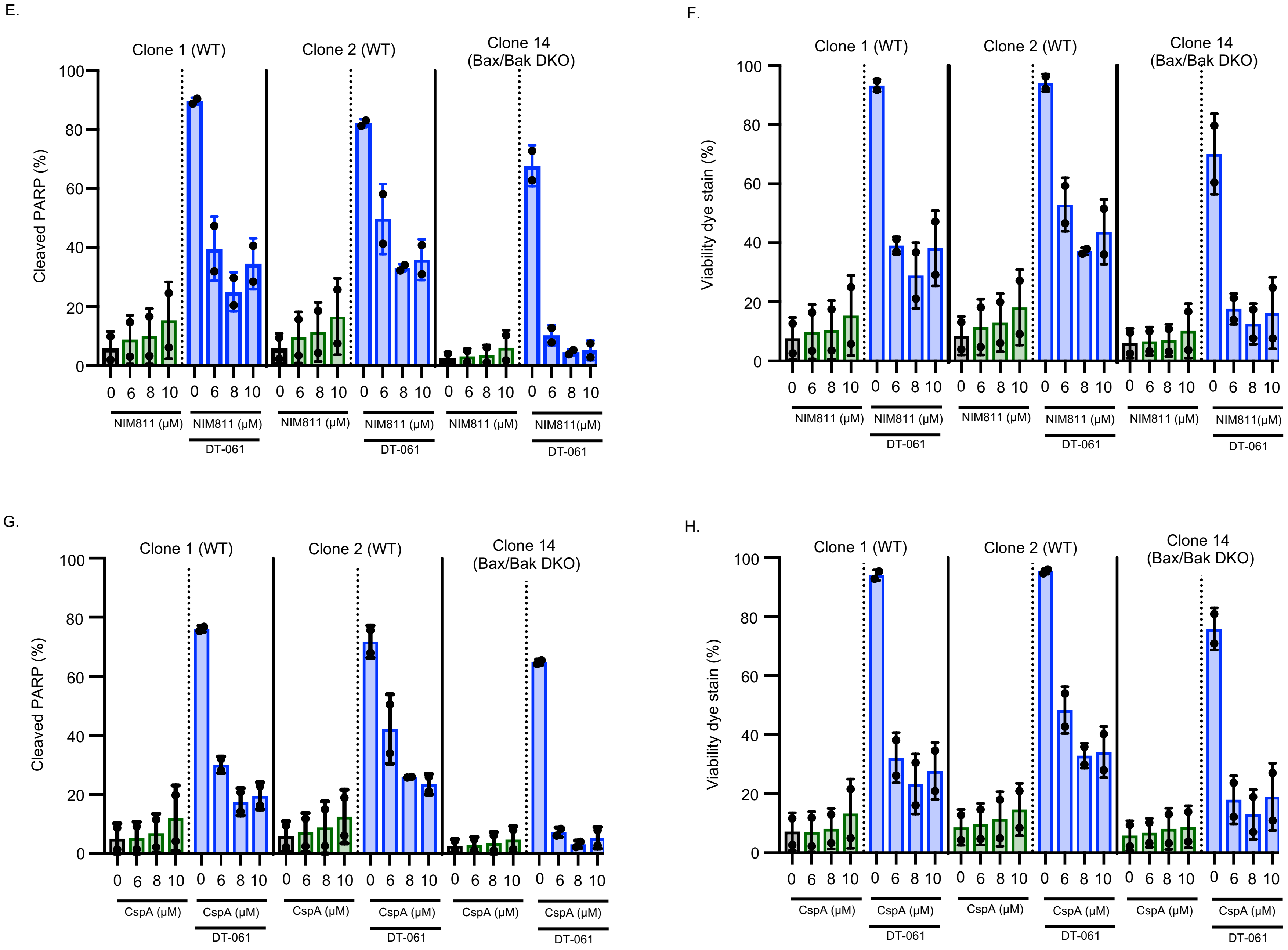
E.
Clone 1 (WT)
Clone 2 (WT)
Clone 14 (Bax/Bak DKO)
Cleaved PARP (%)
0 20 40 60 80 100
0 6 8 10
NIM811 (μM)
NIM811 (μM)
DT-061
NIM811(μM)
F.
Viability dye stain (%)
G.
CspA (μM)
H.

Figure S8

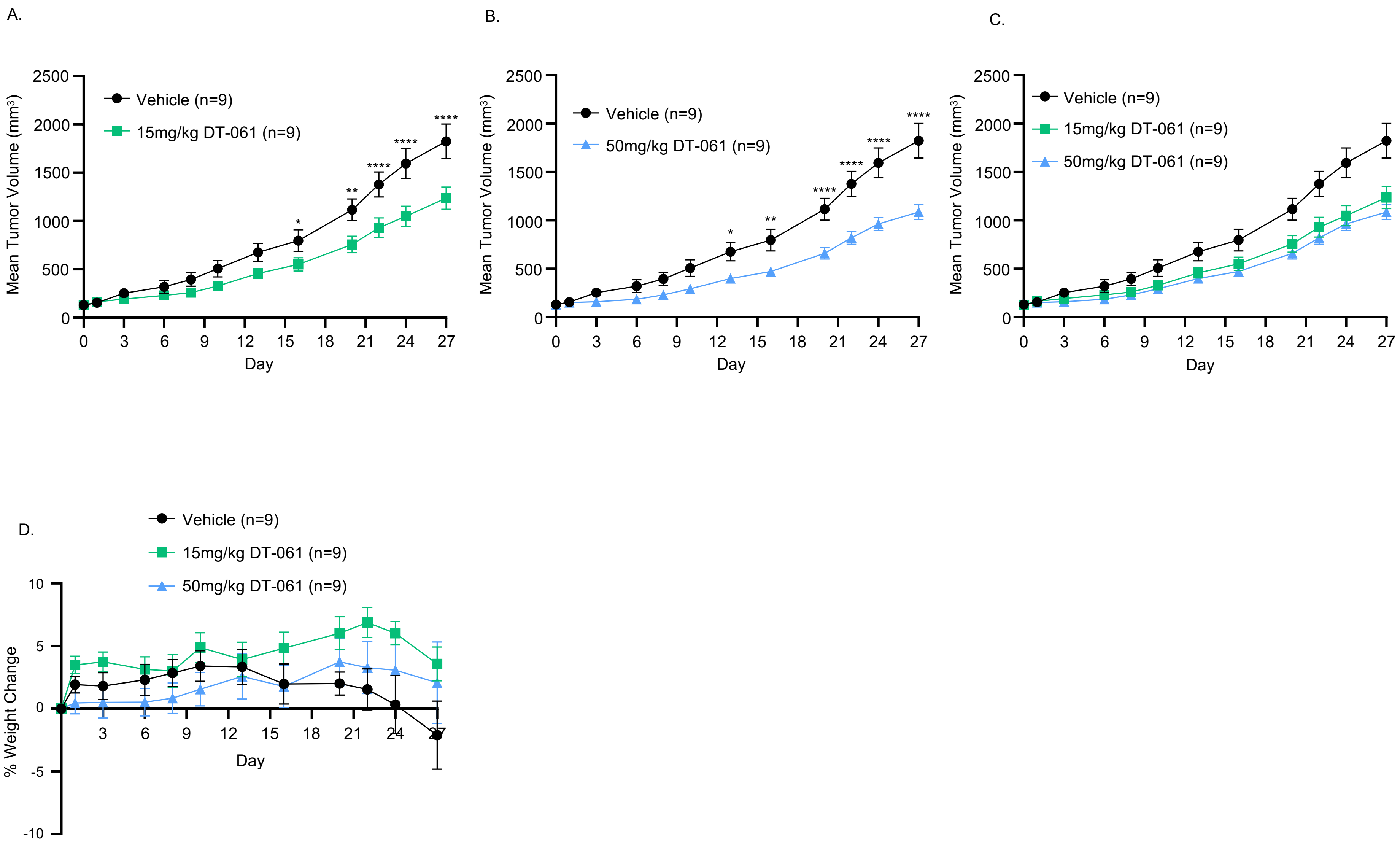
A.
B.
C.
D.
Mean Tumor Volume (mm³)
Day
Vehicle (n=9)
15mg/kg DT-061 (n=9)
50mg/kg DT-061 (n=9)
% Weight Change
*
**
****

Figure S9

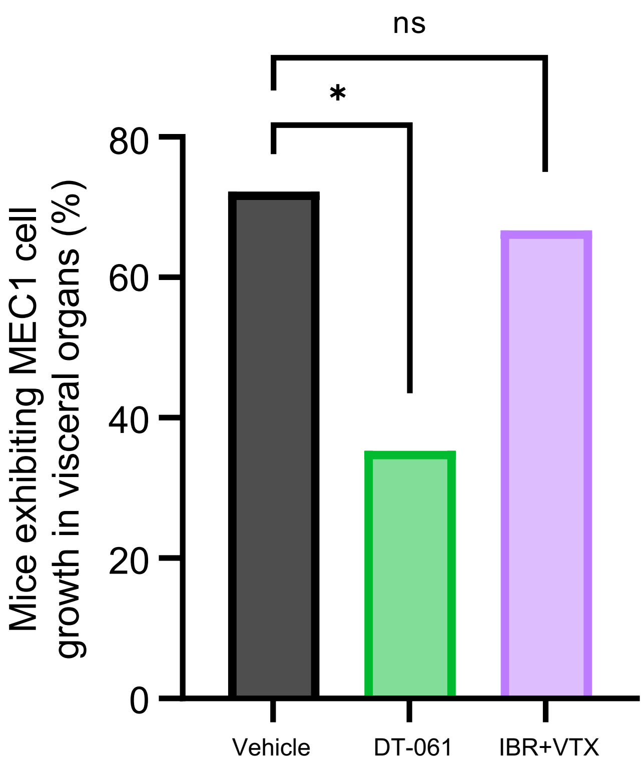

Total number of mice	18	17	18
No. of mice with visible MEC1 cell tumors in visceral organs	13	6	12
Percent mice with visible MEC1 cell tumors in visceral organs	72%	35%	67%

Supplementary figure legends.

Figure S1. CLL samples pre-treated with microenvironmental agonists that induce overexpression of multiple anti-apoptotic proteins exhibit apoptosis resistance due to defective activation of Bax/Bak. (A) Diagram showing experimental approach for analysis of apoptosis resistance in CLL samples *ex vivo*: CLL patient PBMCs were pre-incubated with the combination of CpG-ODN (1.5 µg/ml), sCD40L (1.5 µg/ml), and IL10 (15 ng/ml) ("agonist mix") or mock for 12h and treated with various pro-apoptotic agents as well as second dose of agonist mix for 24h as described in the Methods section. **(B-D)** Samples were treated with inhibitor of Bcl-2 (venetoclax- 6.25, 12.5, 25 nM), Mcl-1 (S63845- 44.7, 105, 176 nM), or Bcl-xL (A1155463- 2, 4, 8 µM) or chemotherapy agents that induce apoptosis (bendamustine- 13.75, 27.48, 54.9 µM; fludarabine- 104, 208, 416 µM; or vincristine- 3.125, 12.5, 50 nM), as described in Panel A. Cleaved PARP was analyzed by flow cytometry. **(B)** Flow cytometry images showing cleaved-PARP in CLL ($CD5^+/CD19^+$) cells in a patient sample (Pt #01) treated with venetoclax (25 nM), S63845 (176 nM), and A1155463 (8 µM) in presence or absence of agonist mix. **(C-D)** Percentage CLL cells showing cleaved-PARP in multiple patient samples treated with various pro-apoptotic agents. **(E-H)** CLL patient PBMCs were treated with venetoclax (VEN, 25 nM), S63845 (176 nM), A1155463 (8 µM), or BTSA1 (100 µM) in presence or absence agonist mix for 18h, as described in Panel A. Bax and Bak activation and cleaved-caspase 9 and -caspase 3 were analyzed in CLL ($CD5^+/CD19^+$) cells by flow cytometry. Activation of Bax and Bak was determined using active conformation specific anti-Bax (clone 6A7) and anti-Bak (clone NT) antibodies, respectively. (E-F) Flow cytometry images showing active Bax (E) or Bak (F) in CLL (CD5+/CD19+) cells in a patient sample treated with VEN in presence or absence of agonist mix. (G-H) Data showing defective activation of Bax along with cleavage of caspase 9 and caspase 3 (G) and defective activation Bak (H) in multiple patient samples. The data in Figure 1C-D and 1G-H are presented after subtracting spontaneous apoptosis values from DMSO treatment controls. Statistical significance was determined by ANOVA with Sidak's post-hoc test for multiple comparisons. *p<0.05, **p<0.01, ***p<0.001, ****p<0.0001, ns- not significant. Data is presented as mean ± SD.

Figure S2. Cleaved caspase 9 analysis in $CD69^{Pos}$ and $CD69^{Neg}$ CLL cells following treatment with anti-apoptotic protein inhibitors. Freshly frozen PBMCs from various CLL patients (Pt #07, 08, 27, 33, 41, 52) were screened in apoptosis threshold assay by incubating

with inhibitor of Bcl-2, Mcl-1, or Bcl-xL for 3h without added agonists as described in Figure 1A-C. Data showing percentage CD69^{Pos} or CD69^{Neg} CLL cells positive for cleaved-caspase 9 from multiple patient samples exposed to various pro-apoptotic agents in apoptosis threshold assay. Statistical significance was determined by ANOVA with Sidak's post-hoc test for multiple comparisons. **p<0.01, ****p<0.0001. Data is presented as mean ± SD.

Figure S3. Development of ibrutinib and venetoclax combination resistant MCL cell lines. (A) Diagram describing the development of drug resistant cell lines. Briefly, MCL cell lines were exposed to DMSO (vehicle) or increasing concentrations of IBR and VEN combination in culture as indicated. Resistant lines were isolated when cells were no longer sensitive to the combination of IBR and VEN at 5 μM dose. **(B-D)** Sensitive and resistant cell lines were treated with IBR, VEN, or the combination of IBR and VEN for 24h. Cell survival was determined by using alamarBlue assay. The data was confirmed in multiple independent experiments as indicated. Statistical significance was determined by ANOVA with Sidak's post-hoc test for multiple comparisons. *p<0.05, **p<0.01, ***p<0.001, ****p<0.0001, ns- not significant. Data is presented as mean ± SD.

Figure S4. Analysis of PP2A activation in DT-061 treated CLL cells. (A) The PP2A activation as assed by analyzing L309 methylation in catalytic subunit of PP2A, as described earlier.(1) MEC1 cells were treated with DMSO or DT-061 (16, 20, 24, and 28 μM) for 3h. The cell lysates were probed for methylated-PP2A(1) and total PP2A by Western blot, as described in Methods. The Western blot image displaying the expression of methylated-PP2A and total-PP2A (top panel). The bar graph chart displaying fold difference in the expression of methylated-PP2A between DMSO and DT-061 treatments (bottom panel). The methylated-PP2A expression was normalized to total PP2A. **(B-C)** MEC1 cells were preincubated with PP2A inhibitor calyculin A (5 nM) or vehicle for 30 min. Then, cells were treated with DMSO or DT-061 (12, 16, or 20 μM) for 3h. Expression of pAKT (S473), pAKT (T308), or total AKT was analyzed by WB. Representative WB image displaying the expression of phosphorylated and total-AKT (top panel). S4B total AKT is the same as S4C total AKT. The bar graph chart displaying fold difference in the expression of methylated-AKT between DMSO and DT-061 treatments (bottom panel). The phosphorylated-AKT expression was normalized to total AKT. Data was confirmed in multiple independent experiments as indicated. Data is presented as mean ± SD.

Figure S5. The PP2A activation by small molecule agonist (DT-061) induces Bax/Bak independent apoptosis in drug resistant CLL cells. (A) The dose response analysis for DT-061 was performed in MEC1 clone1 (WT), clone 2 (WT), and clone 14 (Bax/Bak DKO) cell line. The cytotoxicity was analyzed by alamarBlue assay at 24h of drug treatment. The IC_{50} value was calculated using Prism software. **(B)** The wild type and Bax/Bak DKO MEC1 clones were treated with DT-061 (12, 16, and 20 μM) or combination of VEN (0.2 μM), S63845 (2 μM), and A1155463 (1.6 μM) (SVA) for 12h. The viability dye staining was analyzed by flow cytometry. The average data from three independent experiments presented as bar graph showing percentage cells positive for viability dye. Statistical significance was determined by ANOVA with Sidak's post-hoc test for multiple comparisons. **p<0.01, ***p<0.001, ****p<0.0001, ns- not significant. Data is presented as mean ± SD.

Figure S6. The cell death pathway screening using small molecule inhibitors identifies mPTP as a potential target for SMAP induced cytotoxicity. MEC1 clone 1 (WT), clone2 (WT), and clone 14 (Bax/Bak DKO) cell lines were pre-incubated for 1h with inhibitors targeting various cellular pathways (indirubin and SB216763- GSK3β inhibitors; dantrolene-RyR inhibitor; N-acetyl-L-cysteine and mito-TEMPO -inhibitor of reactive oxygen species or oxidative stress; NIM811 and cyclosporin A- Cyclophilin D inhibitor). Cells were treated with DT-061 (16 μM) for 18h. The cytotoxicity was determined using alamarBlue assay. The data was confirmed in two independent experiments. The average cell survival values are presented as a heatmap.

Figure S7. PP2A modulation by DT-061 activates apoptosis in CLL cells by inducing permeability transition pores in the mitochondria. (A-B) CLL patient samples pre-treated with increasing concentrations of mPTP inhibitor NIM811 for 1h were incubated DT-061 (16 μM) or VEN (25 nM) for 12h. The cleaved-PARP as a readout for apoptosis and viability dye staining as a readout for cell death were analyzed in CLL ($CD5^+/CD19^+$) cells by flow cytometry. Data is presented as bar graphs showing percentage CLL cells positive for cleaved-PARP or viability dye stain. **(C-D)** CLL patient samples pre-treated with increasing concentrations of mPTP inhibitor CspA for 1h were incubated DT-061 (16 μM) or VEN (25 nM) for 12h. The cleaved-PARP and viability dye staining in CLL ($CD5^+/CD19^+$) cells was analyzed by flow cytometry. Data is presented as bar graphs showing percentage CLL cells positive for cleaved-PARP or viability dye stain. **(E-H)** Cyclophilin D inhibitor screening in wild type and Bax/Bak DKO MEC1 cell line. Cells were pre-treated with NIM811 (E-F) or CspA (G-H) for 1h were

incubated DT-061 (20 µM) for 12h. The cleaved PAPR and viability dye staining was analyzed by flow cytometry. Statistical significance was determined by ANOVA with Sidak's post-hoc test for multiple comparisons. *p<0.05, **p<0.01, ***p<0.001, ****p<0.0001. Data is presented as mean ± SD.

Figure S8. DT-061 dose finding study in CLL xenograft mouse model. (A-C) Tumor growth in mice subcutaneously inoculated with MEC-1 cell line and treated with vehicle or DT-061 at two doses. Panel A demonstrates tumor inhibition with DT-061 treatment at 15 mg/kg dose and Panel B demonstrates tumor inhibition with DT-061 at 50 mg/kg. Panel C is presented for comparison of tumor inhibition at two different doses of DT-061. **(D)** The percent body weight change during drug treatment. Data is presented as means ± SEM. Statistical significance was determined by ANOVA with Dunnet's post-hoc. * p < 0.05, ** p < 0.01; ****, p < 0.0001. BID, twice per day; mpk, mg/kg.

Figure S9. DT-061 significantly suppresses tumor growth in tissue microenvironment in vivo in CLL xenograft mouse model. MEC1 cells migrate from the site of inoculation into multiple visceral organs in mice over a period of time(2). The presence of MEC1 cell tumors in liver, kidney, and or spleen were visually examined at the time of sacrifice (end of the study) in MEC1 wild-type xenograft mice treated with vehicle, DT-061 (15 mg/kg), or the combination of IBR and VEN (25 mg/kg). Data is presented as a percentage of mice displaying visible tumors. Statistical significance was determined by Chi-square analysis. *p < 0.05.

Supplementary Tables.

Table S1. Drugs, antibodies, reagents, mouse used in the study.

Drugs/Antibodies/reagents	Source (Company)
Venetoclax	Active Biochem, Maplewood, NJ, USA.
Venetoclax and Ibrutinib for animal study	Selleck, Houston, TX, USA
S63845	Active Biochem Ltd, Hong Kong.
A1155463	APExBIO, TX, USA
NIM811	MCE MedChemExpress, NJ, USA

cyclosporin A, dantrolene, NAC, indirubin, SB216763, mito-TEMPO	Cayman chemical, MI, USA
CpG oligodeoxynucleotides 2006	InvivoGen, San Diego, CA, USA.
Recombinant human IL-10	PeproTech, Rocky Hill, NJ, USA.
Soluble human CD40L	GenScript, Piscataway, NJ. USA.
Protein A/G coated magnetic beads	Pierce Biotechnology, Rockford, IL
Mouse IgG blocking antibody for FACS analysis	Fitzgerald Industries, Acton, MA, USA.
Anti-rabbit (H+L)-AF488, Anti-rabbit (H+L)-AF594	Invitrogen, Eugene, OR, USA
Antibodies: Anti-CD5-APC (clone UCHT2), anti-CD5-APC/CY7 (clone UCHT2), anti-CD19-BV421 (clone HIB19), anti-cleaved PARP-PE or FITC (clone F21-852), anti-RAN (catalogue number- 610341), and anti-Bax (clone 6A7)	BD Biosciences, San Jose, CA, USA.
Anti-CD69-BV605 (clone FN50)	Biolegend, San Diego, CA, USA
Antibodies: Anti-Bcl-xL (clone 54H6), anti-Mcl1 (clone D2W9E), anti-Bcl2 (clone 124), anti-BIM (clone C34C5), and anti-cleaved caspase-9 (Asp315) (clone D8I9E).	Cell Signaling Technologies, Danvers, MA, USA.
Anti-methyl L309 PP2A antibody	Generated by Goutham Narla at the University Michigan(1)
Anti-PP2A antibody (catalytic subunit) (catalogue number- ab106262)	Abcam, Waltham, MA, USA
Anti-Bak (clone NT) (catalogue number- #06-536)	EMD Millipore Corporation, Temecula, CA, USA
Live/Dead viability stains (far infrared or Aqua)	Invitrogen, Eugene, OR, USA

eBioscience Perm/Wash buffer (10X)	Invitrogen, Eugene, OR, USA
Mouse for xenograft study<br><br>-Mouse strain: 014593 C-,129S4-Rag2tm1.1FlvIl2rgtm1.1Flv/J<br>-Genotype: HOM-Rag2 HEM-Il2rg<br>-Gender: Mixed male/female, 70 males and 40 females used in the study (Figure 6)<br>-Age: 6-8 Weeks	Jackson Labs
MEC1 Cell line	Deutsche Sammlung von Mikroorganismen und Zellkulturen GmbH (DSMZ), Germany

Table S2. Clinical characteristics and treatment history of CLL patients.

Pt. No.	Age	Gender	Diagnosis	Previous therapy	FISH studies	Experiments conducted
01	68	M	CLL	FR, ofatumumab	Del13q	Figure 1B (Bax activation)
02	80	F	CLL	Never treated	Trisomy 12	Figure 1B (Bax activation)
03	84	F	CLL	Chlorambucil	Del13q	Figure 1B (Bax activation)
04	75	M	CLL	R-CVP, FR, BR, ofatumumab	Trisomy 12	Figure 3A-B, 4A
07	81	M	CLL	Never treated	Trisomy 12	Figure 1C (Bak activation)
08	69	F	CLL	Never treated	Del13q	Figure 1B (Bax activation)<br>Figure 1C (Bak activation)
14	83	M	CLL	BR, chlorambucil	Unknown	Figure 3A-B, 4A

25	68	M	CLL	Never treated	Del13q	Figure 1B (Bax activation)
27	66	F	CLL	rituximab	Del13q	Figure 1C (Bak activation)
33	63	M	CLL	Obinutuzumab plus bendamustine	Complex	Figure 3A-B, 4A
41	65	M	CLL	Bendamustine, rituximab	Del13q	Figure 1B (Bax activation)<br>Figure 1C (Bak activation)
46	68	M	CLL	Bendamustine, rituximab, IBR	Complex including Del 17p	Figure 4A
52	61	M	CLL	Never treated	Normal	Figure 1B (Bax activation)<br>Figure 1C (Bak activation)
56	56	F	CLL	Never treated	Del13q	Figure 1B (Bax activation)
64	61	F	CLL	FR, BR, IBR	Del13q, Del 17p	Figure 1B (Bax activation)
70	77	F	CLL	Chlorambucil and obinutuzumab	Trisomy12, del17p	Figure 1B (Bax activation)
75	48	F	CLL	Zanubrutinib	Del13q, Del17p	Figure 1B (Bax activation)
80	79	F	CLL	Never treated	Normal	Figure 1B (Bax activation)
81	81	M	CLL	Never treated	Del13q	Figure 1B (Bax activation)
89	39	F	CLL	Never treated	Trisomy14 or disruption	Figure 4A

					of IGH	
95	51	M	CLL	Never treated	Del11q	Figure 1B (Bax activation)
107	67	F	CLL	Never treated	Del 13q	Figure 4A
108	59	M	CLL	FCR, BR, IBR, Acalabrutinib, Obinutuzumab	Del 13q, Del11q	Figure 4A

Abbreviations: IBR, ibrutinib; BR, bendamustine and rituximab; FCR, fludarabine, cyclophosphamide and rituximab; FR, fludarabine and rituximab; N/A, not available.

Table S3. Clinical characteristics and treatment history of CLL patients treated with venetoclax.

Patient No.	FISH studies	Circulating CLL ($CD5^+/CD19^+$) cells (%)		Absolute lymphocyte count (per µl)		Treatment history prior to VEN therapy	Clinical response to VEN treatment
		Pre VEN	During VEN	Pre VEN	During VEN		
12	Del13q	92.2	35.0	47290	5580	BR, Ofatumumab, CP, IBR	CR
21	Del11q, Trisomy 12	40.8	2.1	1580	2310	IBR	CRi
29	Del17p, Trisomy 12	5.2	1.5	1020	1020	Fludarabine, CP, R-CVP, Ofatumumab, IBR, Rituximab	CRi

31	N/A	9.67	1.31	1880	1020	Rituximab, CP, Obinutuzumab, IBR,	CR
71	Del17p	57.2	43.4	56890	27990	IBR	CR

Abbreviations: VEN, venetoclax; IBR, ibrutinib; BR, bendamustine and rituximab; CP, chlorambucil and prednisone; R-CVP, rituximab, cyclophosphamide, vincristine, and prednisone; CR, complete response; CRi, complete response with incomplete marrow recovery. Clinical response to venetoclax treatment was assessed based on the criteria set by the International Workshop on Chronic Lymphocytic Leukemia (iwCLL)(3).

Table S4. SMAP (TRC-382) cytotoxicity analysis in cancer cell lines.

Number	Cell line name	Cancer type	IC_{50} Value	Rank
1	SW13	Adrenal Gland	1.17E+01	20
2	NCIH295R	Adrenal Gland	3.48E+01	227
3	BFTC905	Bladder	1.43E+01	56
4	TCCSUP	Bladder	1.66E+01	93
5	UMUC3	Bladder	1.70E+01	99
6	SCABER	Bladder	1.80E+01	119
7	5637	Bladder	1.83E+01	126
8	HT1197	Bladder	1.86E+01	136
9	639V	Bladder	1.91E+01	144
10	T24	Bladder	1.92E+01	146
11	J82	Bladder	2.26E+01	182
12	647V	Bladder	2.27E+01	183
13	HT1376	Bladder	2.61E+01	198
14	HOS	Bone	1.42E+01	53
15	KHOS240S	Bone	1.62E+01	83
16	U2OS	Bone	1.75E+01	108
17	MG63	Bone	1.82E+01	124
18	SAOS2	Bone	1.92E+01	147

19	SW1353	Bone	2.05E+01	165
20	SJSA1	Bone	2.30E+01	187
21	AU565	Breast	1.16E+01	18
22	T47D	Breast	1.20E+01	22
23	KPL1	Breast	1.29E+01	38
24	MDAMB231	Breast	1.45E+01	61
25	SKBR3	Breast	1.48E+01	63
26	MT3	Breast	1.54E+01	73
27	MDAMB468	Breast	1.57E+01	77
28	MDAMB453	Breast	1.85E+01	133
29	BT474	Breast	1.90E+01	142
30	CAMA1	Breast	1.93E+01	148
31	MCF7	Breast	1.98E+01	156
32	MDAMB436	Breast	2.16E+01	172
33	EFM19	Breast	2.17E+01	175
34	BT20	Breast	2.47E+01	193
35	BT549	Breast	2.96E+01	205
36	HS578T	Breast	3.59E+01	230
37	BE2C	Central Nervous System	1.27E+01	33
38	H4	Central Nervous System	1.27E+01	34
39	MCIXC	Central Nervous System	1.30E+01	39
40	U87MG	Central Nervous System	1.54E+01	74
41	CHP212	Central Nervous System	1.72E+01	101
42	SKNAS	Central Nervous System	1.77E+01	113

43	A172	Central Nervous System	1.83E+01	127
44	D283MED	Central Nervous System	1.97E+01	155
45	SW1088	Central Nervous System	2.17E+01	176
46	DAOY	Central Nervous System	2.18E+01	177
47	SNB19	Central Nervous System	2.29E+01	185
48	SKNFI	Central Nervous System	2.44E+01	192
49	U138MG	Central Nervous System	2.64E+01	199
50	SW1783	Central Nervous System	2.84E+01	203
51	T98G	Central Nervous System	2.89E+01	204
52	CCFSTTG1	Central Nervous System	3.17E+01	212
53	DBTRG05MG	Central Nervous System	3.20E+01	215
54	SKNDZ	Central Nervous System	3.36E+01	221
55	DKMG	Central Nervous System	3.93E+01	234
56	Y79	Eye	3.20E+01	216
57	HEC1A	Female GU	1.20E+01	23
58	HELA	Female GU	1.21E+01	26
59	CAOV3	Female GU	1.22E+01	27
60	JAR	Female GU	1.32E+01	41

61	JEG3	Female GU	1.45E+01	62
62	C4II	Female GU	1.49E+01	68
63	ES2	Female GU	1.59E+01	78
64	SW954	Female GU	1.62E+01	84
65	C4I	Female GU	1.66E+01	94
66	OVCAR3	Female GU	1.74E+01	105
67	HT3	Female GU	1.77E+01	114
68	AN3CA	Female GU	1.78E+01	117
69	DOTC24510	Female GU	1.93E+01	149
70	RL952	Female GU	1.93E+01	150
71	SIHA	Female GU	1.99E+01	158
72	BEWO	Female GU	2.51E+01	194
73	C33A	Female GU	3.21E+01	217
74	KLE	Female GU	3.39E+01	222
75	SW962	Female GU	3.75E+01	232
76	SKOV3	Female GU	3.96E+01	235
77	FADU	Head and Neck	1.42E+01	54
78	CAL27	Head and Neck	1.48E+01	64
79	DETROIT562	Head and Neck	1.60E+01	80
80	OE19	Head and Neck	1.62E+01	85
81	SCC25	Head and Neck	1.65E+01	92
82	OE33	Head and Neck	1.81E+01	122
83	A431	Head and Neck	1.85E+01	134
84	OE21	Head and Neck	1.99E+01	159
85	SCC4	Head and Neck	2.96E+01	206
86	SCC9	Head and Neck	3.15E+01	211
87	G402	Kidney	9.91E+00	10
88	769P	Kidney	1.42E+01	55
89	SKNEP1	Kidney	1.49E+01	69
90	CAKI2	Kidney	1.52E+01	70

91	G401	Kidney	1.59E+01	79
92	A498	Kidney	1.66E+01	95
93	ACHN	Kidney	1.67E+01	98
94	CAKI1	Kidney	1.77E+01	115
95	786O	Kidney	1.83E+01	128
96	RKO	Large Intestine	9.91E+00	11
97	RKOE6	Large Intestine	1.15E+01	15
98	RKOAS451	Large Intestine	1.19E+01	21
99	HCT116	Large Intestine	1.24E+01	28
100	HCT8	Large Intestine	1.27E+01	35
101	HCT15	Large Intestine	1.34E+01	43
102	DLD1	Large Intestine	1.36E+01	46
103	WIDR	Large Intestine	1.41E+01	52
104	HT29	Large Intestine	1.44E+01	58
105	COLO320DM	Large Intestine	1.48E+01	65
106	COLO320HSR	Large Intestine	1.62E+01	86
107	SW48	Large Intestine	1.66E+01	96
108	LS174T	Large Intestine	1.72E+01	102
109	COLO205	Large Intestine	1.74E+01	106
110	COLO201	Large Intestine	1.75E+01	109
111	LS1034	Large Intestine	1.84E+01	131
112	SW620	Large Intestine	1.95E+01	152
113	SW480	Large Intestine	2.03E+01	160
114	SW837	Large Intestine	2.16E+01	173
115	NCIH747	Large Intestine	2.18E+01	178
116	SW1417	Large Intestine	2.29E+01	186
117	NCIH508	Large Intestine	2.60E+01	197
118	SW1463	Large Intestine	3.18E+01	213
119	SW948	Large Intestine	3.49E+01	228
120	SW403	Large Intestine	4.00E+01	236

121	MOLT16	Leukemia	6.24E+00	1
122	CCRFCEM	Leukemia	6.69E+00	2
123	CEMC1	Leukemia	7.22E+00	3
124	CMLT1	Leukemia	8.02E+00	5
125	MX1	Leukemia	9.61E+00	9
126	BV173	Leukemia	1.26E+01	31
127	MHHPREB1	Leukemia	1.33E+01	42
128	JRT3T35	Leukemia	1.36E+01	47
129	JURKAT	Leukemia	1.53E+01	71
130	NALM6	Leukemia	1.64E+01	91
131	K562	Leukemia	1.77E+01	116
132	HEL9217	Leukemia	1.80E+01	120
133	MV411	Leukemia	1.86E+01	137
134	EM2	Leukemia	2.04E+01	162
135	MEG01	Leukemia	2.18E+01	179
136	MOLT3	Leukemia	2.80E+01	202
137	THP1	Leukemia	3.35E+01	220
138	HLE	Liver	1.20E+01	24
139	HUH6CLONE5	Liver	1.28E+01	36
140	SNU423	Liver	1.72E+01	103
141	HLF	Liver	1.83E+01	129
142	OCUG1	Liver	2.09E+01	167
143	HUCCT1	Liver	2.51E+01	195
144	HEPG2	Liver	3.31E+01	218
145	CALU6	Lung	1.31E+01	40
146	A427	Lung	1.34E+01	44
147	DMS273	Lung	1.36E+01	48
148	CHAGOK1	Lung	1.40E+01	51
149	NCIH661	Lung	1.48E+01	66
150	CORL105	Lung	1.62E+01	87

151	CORL23	Lung	1.63E+01	88
152	NCIH460	Lung	1.63E+01	89
153	A549	Lung	1.73E+01	104
154	SKMES1	Lung	1.81E+01	123
155	NCIH292	Lung	1.82E+01	125
156	SHP77	Lung	1.87E+01	139
157	NCIH520	Lung	1.98E+01	157
158	DMS114	Lung	2.11E+01	169
159	NCIH446	Lung	2.13E+01	170
160	NCIH69	Lung	2.65E+01	200
161	DMS53	Lung	2.66E+01	201
162	CALU1	Lung	3.33E+01	219
163	SW900	Lung	3.54E+01	229
164	WI38	Lung	3.64E+01	231
165	NCIH596	Lung	3.87E+01	233
166	NCIH441	Lung	4.00E+01	237
167	RAJI	Lymphoma	7.98E+00	4
168	DOHH2	Lymphoma	8.87E+00	6
169	ST486	Lymphoma	9.38E+00	8
170	SR	Lymphoma	1.08E+01	14
171	BC1	Lymphoma	1.15E+01	16
172	CROAP2	Lymphoma	1.15E+01	17
173	RAMOSRA1	Lymphoma	1.26E+01	32
174	DB	Lymphoma	1.44E+01	59
175	RPMI6666	Lymphoma	1.60E+01	81
176	HT	Lymphoma	1.61E+01	82
177	L428	Lymphoma	1.84E+01	132
178	EB3	Lymphoma	2.06E+01	166
179	DAUDI	Lymphoma	2.16E+01	174
180	RPMI8226	Myeloma	1.43E+01	57

181	U266B1	Myeloma	1.44E+01	60
182	ARH77	Myeloma	1.54E+01	75
183	SKO007	Myeloma	1.86E+01	138
184	HPAFII	Pancreas	9.06E+00	7
185	MIAPACA2	Pancreas	1.53E+01	72
186	CAPAN2	Pancreas	1.83E+01	130
187	SU8686	Pancreas	1.85E+01	135
188	HS766T	Pancreas	1.87E+01	140
189	ASPC1	Pancreas	1.90E+01	143
190	CFPAC1	Pancreas	2.10E+01	168
191	YAPC	Pancreas	2.14E+01	171
192	PANC1	Pancreas	2.23E+01	180
193	BXPC3	Pancreas	2.35E+01	188
194	HUPT4	Pancreas	2.41E+01	190
195	CAPAN1	Pancreas	4.00E+01	238
196	22RV1	Prostate	1.56E+01	76
197	LNCAP	Prostate	1.66E+01	97
198	BM1604	Prostate	1.78E+01	118
199	PC3	Prostate	1.89E+01	141
200	DU145	Prostate	1.95E+01	153
201	BPH1	Prostate	4.00E+01	239
202	A375	Skin (Melanoma)	1.06E+01	12
203	A7	Skin (Melanoma)	1.07E+01	13
204	HMCB	Skin (Melanoma)	1.28E+01	37
205	CHL1	Skin (Melanoma)	1.75E+01	110
206	COLO829	Skin (Melanoma)	1.80E+01	121
207	A101D	Skin (Melanoma)	1.93E+01	151
208	SKMEL28	Skin (Melanoma)	1.95E+01	154
209	MEWO	Skin (Melanoma)	2.04E+01	163
210	SH4	Skin (Melanoma)	2.04E+01	164

211	HS695T	Skin (Melanoma)	2.24E+01	181
212	SKMEL3	Skin (Melanoma)	2.42E+01	191
213	RPMI7951	Skin (Melanoma)	2.59E+01	196
214	SKMEL1	Skin (Melanoma)	3.11E+01	208
215	C32TG	Skin (Melanoma)	3.14E+01	209
216	HS294T	Skin (Melanoma)	3.19E+01	214
217	MALME3M	Skin (Melanoma)	3.39E+01	223
218	C32	Skin (Melanoma)	3.44E+01	225
219	A204	Soft Tissue	1.16E+01	19
220	RD	Soft Tissue	1.20E+01	25
221	SW872	Soft Tissue	1.25E+01	29
222	MESSA	Soft Tissue	1.37E+01	49
223	SJRH30	Soft Tissue	1.39E+01	50
224	SKUT1	Soft Tissue	1.48E+01	67
225	TE381T	Soft Tissue	1.74E+01	107
226	SKLMS1	Soft Tissue	1.76E+01	111
227	HT1080	Soft Tissue	1.91E+01	145
228	SW982	Soft Tissue	2.36E+01	189
229	A673	Soft Tissue	3.14E+01	210
230	SW684	Soft Tissue	4.00E+01	240
231	AGS	Stomach	1.25E+01	30
232	SNU16	Stomach	1.35E+01	45
233	SNU1	Stomach	1.63E+01	90
234	KATOIII	Stomach	1.70E+01	100
235	HS746T	Stomach	3.00E+01	207
236	SNU5	Stomach	3.43E+01	224
237	CAL62	Thyroid	1.76E+01	112
238	CGTHW1	Thyroid	2.03E+01	161
239	SW579	Thyroid	2.28E+01	184
240	BHT101	Thyroid	3.46E+01	226

Cell line screen for TRC-382 (Figure 3A) was performed by Eurofins/Pans Lab as described in our earlier paper(4).

Table S5. Information of sgRNAs used in the Bax/Bak double knockout MEC1 cell line development.

Target gene	sgRNA Sequence
BAX	CACCGTTTCTGACGGCAACTTCAAC
	AAACGTTGAAGTTGCCGTCAGA AAC
	CACCGAGTAGAAAAGGGCGACAACC
	AAACGGTTGTCGCCCTTTTCTACTC
BAK1	CACCGACGGCAGCTCGCCATCATCG
	AAACCGATGATGGCGAGCTGCC GTC
	CACCGTTGATGTCGTCCCCGATGA
	AAACTCATCGGGGACGACATCAAC
	CAC CGCTCACCTGCTAGGTTGCAG
	AAACCTGCAACCTAGCAGGTGAGC

Supplementary Methods.

The analysis of drug induced cytotoxicity and Bax/Bak activation by flow cytometry.

To induce CLL cell activation (Figure S1), patient PBMC samples were cultured with agonists [CpG-ODN (1.5 μg/ml)+sCD40L (2 μg/ml)+IL-10 (15 ng/ml); "agonist-mix"] for 12h. Then, a second dose of agonist mix as well as drugs were added to culture as indicated. The Bax/Bak activation (Figure 4A and S1E-H) and drug induced cytotoxicity (Figure 4A, 4D, 5A-B, S1A-D, S5B, and S7) were analyzed using flow cytometry as described earlier(5). Briefly, dead cells were stained by incubating PBMC samples with Live/Dead near-infrared or aqua viability dye for 20 min at 4°C. Then, samples were fixed in 1.6% paraformaldehyde and blocked with mouse IgG. Then, surface proteins were stained by incubating with appropriate antibodies for 1h at 4°C. Intracellular proteins were stained with appropriate antibodies after permeabilizing cells with saponin based permeabilization buffer (eBiosciences, San Jose, CA) at 4°C. Flow cytometry was performed using Attune NXT (Life Technologies) and CytoFLEX S (Beckman Coulter) analytical flow cytometers. Flow cytometry data analysis was done using FlowJo software (version 10.5.3) (Ashland, OR).

Following antibody panels were used for drug induced cytotoxicity or Bax/Bak activation analysis. Figure S1A-D: Live/Dead near-infrared viability stain, anti-CD5-APC, anti-CD19-BV421, and anti-cleaved PARP-PE. Figure S1E and S1G: Anti-CD5-APC/CY7, anti-CD19-BV421, anti-caspase-9 primary antibody followed by anti-rabbit AF594, anti-BAX (clone 6A7)-PE, and anti-cleaved caspase-3-AF647. Figure S1F and S1H: anti-CD5-APC/CY7, anti-CD19-BV421, and anti-BAK (clone NT) primary antibody followed by anti-rabbit AF594. Figure 4A: Live/Dead aqua viability stain, anti-CD5-APC/Cy7, anti-CD19-BV421, anti-BAX (clone 6A7)-PE, anti-cleaved-PARP-FITC, and anti-caspase 9 primary antibody followed by anti-rabbit AF594. Figure 4B: Live/Dead near-infrared viability stain, anti-CD5-APC, anti-CD19-BV421, anti-CD69 BV605, anti-BAX (clone 6A7)-PE, and anti-cleaved PARP-FITC. Figure 4D and S5B: Live/Dead near-infrared viability stain and anti-cleaved-PARP-PE. Figure 5A-B and S7A-D: Live/Dead Aqua viability stain, anti-CD5-APC/CY7, anti-CD19-BV421, anti-caspase 9 primary antibody followed by anti-rabbit AF594, and anti-cleaved-PARP-PE. Figure S7E-H: Live/Dead aqua viability stain and anti-cleaved PARP-PE. Antibody details are in Supplementary Table S1.

Cell viability assay

Cell viability in Figure 3, S3, S5A, and S6 was determined by alamarBlue as per the manufacturer's protocol (Invitrogen). Briefly, cells were cultured with various drugs as indicated for 18h. Then, 10μl of alamarBlue reagent was added to each well and incubated at 37°C for 6h. Fluorescence was measured at 560 nm excitation/590 nm emission on a Synergy 2 plate reader (BioTek Instruments). Mean results and SD were calculated for duplicate samples.

Development of Bax and Bak double knockout CLL cell line MEC1.

Bax and Bak double knockout MEC1 cell line was developed using CRISPR-Cas9 system, as described earlier(6). Multiple guide RNAs (sgRNAs) targeting human Bax and Bak1 genes were selected from the available Addgene CRISPR pooled sgRNA Library. The sgRNAs targeting human BAX and BAK1 (Table S5) were cloned into TLCV2 plasmid backbone (Addgene, #87360) using restriction enzyme BsmBI and confirmed by Sanger DNA sequencing. An empty TLCV2 was included as control. VSV pseudotyped lentiviruses expressing Bax and Bak sgRNAs and Cas9 and GFP proteins were generated by co-transfecting 293T cells with TLCV2 vectors containing pool of sgRNA targeting Bax/Bak and lentivirus packaging plasmids psPAX2 and pMD2. The transfection was performed using Lipofectamine 3000 transfection reagent (Thermo Fisher Scientific, USA). Lentiviruses were concentrated from the supernatants

at 48h post-transfection through ultracentrifugation (125, 000*g* for 3h). The virus titer was determined by analyzing GFP expression in flow cytometry following transduction of Jurkat cells. MEC1 cells were transduced with 100 MOI of lentivirus expressing sgRNAs against Bax and Bak1 genes in presence of Polybrene. After 48h of transduction, cells were selected in presence of puromycin for a week. Subsequently, single cell clones were prepared by serial dilution protocol and Bax/Bak expression loss was confirmed by Western blot. Doxycycline was added during puromycin selection as well as single cell cloning to induce Cas9 and GFP expression.

References.

1. Leonard D, Huang W, Izadmehr S, OConnor CM, Wiredja DD, Wang Z, et al. Selective PP2A Enhancement through Biased Heterotrimer Stabilization. *Cell.* 2020;181(3):688-701.e16.
2. Bertilaccio MT, Scielzo C, Simonetti G, Ponzoni M, Apollonio B, Fazi C, et al. A novel Rag2-/-gammac-/--xenograft model of human CLL. *Blood.* 2010;115(8):1605-9.
3. Hallek M, Cheson BD, Catovsky D, Caligaris-Cappio F, Dighiero G, Döhner H, et al. iwCLL guidelines for diagnosis, indications for treatment, response assessment, and supportive management of CLL. *Blood.* 2018;131(25):2745-60.
4. McClinch K, Avelar RA, Callejas D, Izadmehr S, Wiredja D, Perl A, et al. Small-Molecule Activators of Protein Phosphatase 2A for the Treatment of Castration-Resistant Prostate Cancer. *Cancer Res.* 2018;78(8):2065-80.
5. Jayappa KD, Portell CA, Gordon VL, Capaldo BJ, Bekiranov S, Axelrod MJ, et al. Microenvironmental agonists generate de novo phenotypic resistance to combined ibrutinib plus venetoclax in CLL and MCL. *Blood Adv.* 2017;1(14):933-46.
6. Jayappa KD, Gordon VL, Morris CG, Wilson B, Shetty BD, Cios KJ, et al. Extrinsic interactions in the microenvironment in vivo activate an antiapoptotic multidrug-resistant phenotype in CLL. *Blood Adv.* 2021;5(17):3497-510.
